# A fully human IgG1 antibody targeting MICA α1 domain inhibits interaction with NKG2D and activates immune effector functions against MICA-expressing cells

**DOI:** 10.3389/fimmu.2026.1740184

**Published:** 2026-02-11

**Authors:** Ivo Campos, Fabiola González-Herrera, Yuneisy Guerra, María José Garrido, Nicolás Fehring, Carla Diaz, Douglas J. Matthies, Mauricio González, Samantha Tello, Fabián Tempio, Gonzalo Vásquez, Jose Rodríguez-Siza, Matías Gutiérrez, Lorenzo Leiva, Alfonso Romero, Norberto Collazo, Roberto Zúñiga, Carolina Valck, Carolina H. Ribeiro, Claudia Altamirano, Karen Toledo-Stuardo, María Carmen Molina

**Affiliations:** 1 Núcleo Interdisciplinario de Farmacología e Inmunología, Instituto de Ciencias Biomédicas (ICBM), Facultad de Medicina, Universidad de Chile, Santiago, Chile; 2 Escuela de Ingeniería Bioquímica, Pontificia Universidad Católica de Valparaíso, Valparaíso, Chile; 3 Interventional Medicine for Precision and Advanced Cellular Therapy (IMPACT), Center of Interventional Medicine for Precision and Advanced Cellular Therapy, Santiago, Chile; 4 Centro Regional de Estudios en Alimentos Saludables, Valparaíso, Chile

**Keywords:** ADCC, ADCP, cancer immunotherapy, CDC, full human antibody, MICA, NKG2D

## Abstract

**Background:**

MICA is a stress-induced ligand for the activating receptor NKG2D and plays a central role in immune recognition and elimination of tumor cells. Tumor-derived soluble MICA (sMICA) and its allelic diversity impair immune responses by disrupting NKG2D function and promoting immune evasion, highlighting the need for antibody-based strategies targeting the MICA/NKG2D axis.

**Methods:**

We generated a fully human IgG1 monoclonal antibody targeting the α1 domain of MICA using phage display and rational epitope design focused on a conserved, low-polymorphic region. We characterized its binding specificity and affinity for MICA, competition with NKG2D, and its ability to trigger immune effector functions, including antibody-dependent cell cytotoxicity (ADCC), antibody-dependent cell phagocytosis (ADCP) and activation of the classical complement pathway.

**Results:**

The antibody displayed high affinity for recombinant MICA (app K_d_ ≈ 20 nM) and effectively competed with NKG2D (K_i_ ≈ 15 nM). It recognized multiple MICA alleles expressed in human gastric and leukemia cell lines, confirming broad allelic coverage. Functional assays showed that the antibody mediated strong CDC, ADCC by NK cells and promoted ADCP of MICA-expressing cells.

**Conclusion:**

By combining high-affinity recognition of broad MICA alleles and robust activation of immune effector functions, this fully human anti-MICA α1 antibody offers a novel strategy to modulate the MICA/NKG2D axis and activate Fc-mediated immune effector functions against MICA-expressing tumor cells. Its dual mechanism of action supports its development as a standalone or combinatorial agent with immune checkpoint inhibitors in MICA-expressing tumors.

## 1 Introduction

Major histocompatibility complex class I polypeptide-related sequence A (MICA) is a non-classical MHC class I molecule that plays a vital role in immune surveillance under both healthy and pathological conditions (1–5). Its biological significance arises from engagement to natural killer group 2, member D (NKG2D), an activating receptor expressed on the surface of NK cells and certain T cell subsets, which triggers cytotoxic granule release (perforin, granzymes) and secretion of pro-inflammatory cytokines (6–10). As a stress-inducible protein, MICA acts as a “kill me” signal, which leads to elimination of the target cell.

Structurally, MICA comprises three extracellular domains (α1, α2 and α3) and a transmembrane domain. Together, the α1 and α2 domains constitute the central interface for NKG2D binding, which is crucial for immunological synapse formation and subsequent clearance of tumor or pathogen-infected cells (11, 12).

Under physiological conditions, MICA is found in the cytoplasm (3, 5, 13), on the cell membrane (1, 3–5), or associated with extracellular vesicles (EVs) (14). In the tumor microenvironment, however, malignant cells often evade NKG2D-mediated killing by shedding soluble MICA (sMICA) through the action of metalloproteases, avoiding the recognition of tumor cells (10, 15, 16). These soluble forms of MICA act also as decoys that downregulate NKG2D expression on cytotoxic immune cells, thus impairing their function and favoring tumor progression (17).

Accumulating clinical and translational data position the MICA/NKG2D axis as a key immunoregulatory pathway with implications for tumor progression, prognosis, and therapeutic responsiveness. Aberrant modulation of this axis (particularly the imbalance between membrane-bound and soluble MICA) has been associated with poor clinical outcomes and diminished efficacy of immune checkpoint blockade and adoptive cell therapies (18, 19). High levels of soluble MICA in patient plasma correlate with advanced disease stage and immune dysfunction, whereas preserved NKG2D signaling is linked to improved survival and treatment response across several malignancies (20). Accordingly, endogenous anti-MICA antibodies have been described in Monoclonal Gammopathy of Undetermined Significance patients, which help maintain immune surveillance by neutralizing sMICA and facilitating antigen cross-presentation (21).

These findings have positioned MICA/NKG2D not merely as a biomarker of immune competence but as a clinically actionable axis whose integrity determines the effectiveness of antitumor immunity. This concept provides the biological and translational rationale for developing next-generation immunotherapies aimed at restoring or enhancing MICA-NKG2D interactions.

Notably, MICA exhibits extensive genetic diversity, with more than 300 allelic variants identified to date (https://hla.alleles.org/pages/genes/genes_list/), of which MICA*008 and MICA*002 are among the most prevalent worldwide (http://www.allelefrequencies.net/). Several polymorphisms derive from non-synonymous amino acid substitutions into the α1/α2 domains, thus altering MICA conformation and capacity to bind NKG2D (22). Some alleles also correlate with cancer susceptibility and prognosis, underscoring the clinical importance of MICA allelic variation (4).

Over the past decade, multiple fully human or humanized MICA-specific monoclonal antibodies (mAbs) have been developed (primarily directed against the α3 domain) to block proteolytic shedding of MICA without disrupting NKG2D engagement. For instance, DM919 (NCT06328673), 7C6 (19) and AHA-1031 (23) each recognize the membrane-proximal α3 domain and induce potent antibody-dependent cellular cytotoxicity (ADCC) and antibody-dependent cellular phagocytosis (ADCP), suppressing tumor growth in cholangiocarcinoma, acute myeloid leukemia (24), and KRAS/LKB1-mutant lung cancer (23). In contrast, CLN-619 (NCT05117476) binds conformational epitopes spanning the α1/α2 regions, thus preserving NKG2D interactions while leveraging Fc-mediated effector mechanisms.

Despite these advancements, the α1 domain has received comparatively little attention as a therapeutic target, even though it is essential for NKG2D binding and exhibits lower polymorphism than α2 or α3 domains (8, 25). Here, we report a novel fully human IgG1 mAb specifically directed against MICA α1 domain (anti-MICA c65). By selecting an antigenic region with reduced polymorphism, our antibody recognizes multiple MICA alleles on tumor cells, broadening its therapeutic applicability. Moreover, in addition to blocking the interaction between MICA and NKG2D *in vitro*, our IgG1 format stimulates several Fc-mediated effector functions, including ADCC, ADCP and activation of the classical complement pathway.

Mechanistically, our α1-targeted mAb operates via two complementary axes: first, it blocks the interaction between MICA and its receptor NKG2D, as demonstrated in an *in vitro* binding assay, and second, it recognizes membrane-bound MICA on tumor cells to further activate effector responses through its Fc domain. By addressing challenges related to MICA polymorphism and interference with NKG2D engagement, this α1-domain specific antibody could expand the range of tumors amenable to immunotherapy and improve clinical efficacy in diseases where NKG2D–MICA interactions are pivotal.

## 2 Materials and methods

### 2.1 Ethical considerations

Peripheral blood samples were collected from healthy adult volunteers (≥18 years old) recruited at the Faculty of Medicine, University of Chile. Prior to enrollment, each volunteer provided written informed consent, in accordance with the Declaration of Helsinki and local regulatory requirements. The study protocol was reviewed and approved by the Committee on Ethics in Human Research (CEISH) of the Faculty of Medicine, University of Chile (Project No. 022-2022, Acta No. 002).

### 2.2 Cell lines

K562 chronic myeloid leukemia cell line (ATCC^®^ CCL-243™) was obtained from the American Type Culture Collection (ATCC^®^, USA). GES-1 and MKN-45 gastric cell lines were kindly provided by Dr. Andrew Quest (Biomedical Sciences Institute (ICBM), Faculty of Medicine, University of Chile). Chinese hamster ovary (CHO) cells were provided by Dr. Mar Valés-Gómez (Spanish National Centre for Biotechnology CNB-CSIC). U937-EGFP cell line, derived from human promonocytic myeloid leukemia (ATCC^®^ CRL-1593.2, USA), was treated with 10 ng/mL phorbol 12-myristate 13-acetate (PMA) (Sigma-Aldrich, USA) for 48 h to induce macrophage differentiation, following established protocols (26). Human and hamster cell lines were cultured in RPMI-1640 (Gibco, USA) and DMEM (Gibco, USA), respectively, supplemented with 10% fetal bovine serum (FBS) (Sigma-Aldrich, USA), and 1× penicillin-streptomycin (Gibco, USA), and incubated at 37°C and 5% CO_2_.

### 2.3 Human NK cells isolation

Peripheral blood mononuclear cells (PBMC) were obtained from a total of 6 healthy donors, and cell isolation was performed by a density gradient using Lymphoprep™ density gradient medium (STEMCELL Technologies, Vancouver, Canada). NK cells were purified using a negative selection kit (Human NK Cell Isolation Kit, Miltenyi Biotec, Bergisch Gladbach, Germany), according to the manufacturer’s instructions. NK cells (effector cells) were cultured with 10 ng/mL recombinant human IL-2 (Cell Signaling Technology, Danvers, MA, USA) for 5–6 days before co-cultures with K562 cells (target cells). Cell viability was analyzed with 0.4% trypan blue (Sigma-Aldrich, USA).

### 2.4 Recombinant human proteins

Untagged MICA*001, containing the α1 and α2 domains (residues 1-183) (recombinant MICA), and His-tagged extracellular domain of human NKG2D (residues 80–216) were cloned into the pET-15b vector, transformed into *Escherichia coli* BL21(DE3), and expressed as inclusion bodies following the protocol described by Gutiérrez-González (27). Protein expression was induced with 1 mM IPTG for 3 h at 37°C and 200 rpm. The bacterial cultures were harvested, and the inclusion bodies were extracted and solubilized in a solubilization buffer (50 mM Tris, 500 mM NaCl, 6 M guanidine hydrochloride, 25 mM imidazole, pH 8.0) under gentle shaking overnight at 4°C. The samples were centrifuged at 10, 000 × *g* for 10 min at 4°C. NKG2D was purified under denaturing conditions using affinity chromatography with a Ni-NTA matrix. After incubation with the resin for 2 h at 4°C under gentle agitation, the column was washed twice with the solubilization buffer. Bound proteins were eluted with an elution buffer (50 mM Tris, 500 mM NaCl, 300 mM imidazole, pH 8.0). Protein concentration in the eluted fractions was determined using the Bradford method, and the samples were stored at -20°C, until use. Denatured MICA and NKG2D were refolded *in vitro* overnight at 4°C by a 100-fold dilution using a drop-wise method into a renaturation buffer containing 50 mM Tris, 3 mM reduced glutathione, and 0.3 mM oxidized glutathione, supplemented with 500 mM NaCl and 500 mM L-arginine. The refolded proteins were then centrifuged at 10, 000 × g for 10 min at 4°C, concentrated using a centrifugal filter unit, and buffer-exchanged to PBS (pH 7.4).

### 2.5 Generation of anti-MICA single-chain variable fragments using phage display

The scFv phage display library used in this study was the same library previously generated and characterized by Sotelo et al, (28)constructed using independent strand amplification for variable region assembly. The library was originally generated from PBMCs obtained from healthy donors as described in detail in (28). Briefly, variable heavy (VH) and kappa light (VL) chains were amplified, assembled into a single fragment, using a flexible linker [(Gly_4_Ser)_3_], and cloned in frame with the M13 phage pIII protein, allowing the display of individual human scFv fragments on the surface of filamentous phages.

**Figure 1:**
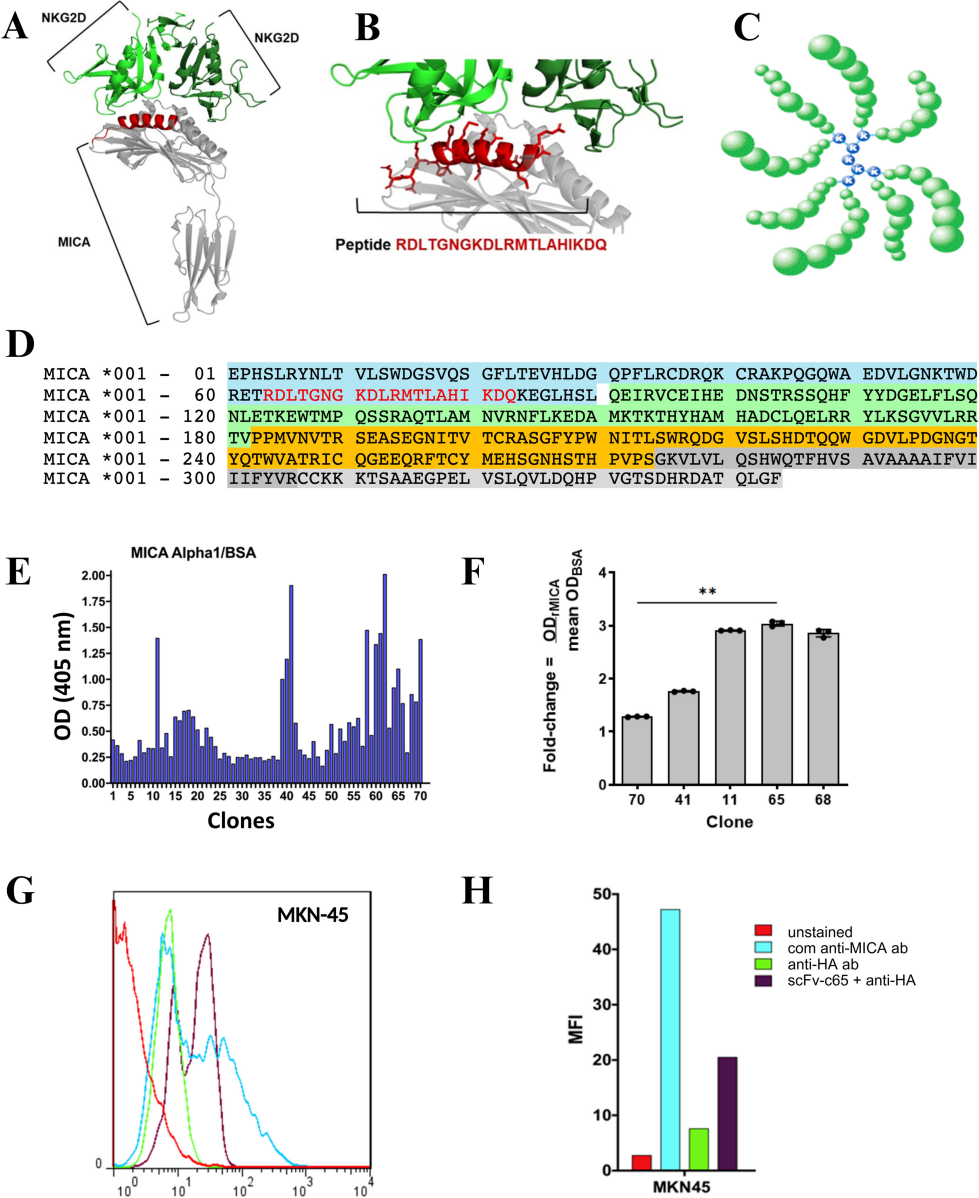
Generation and selection of anti-MICA single-chain variable fragments (scFv) using phage display and epitope-directed design. **(A, B)** Schematic diagrams of the epitope-focused phage display strategy used to generate human anti-MICA scFv clones, targeting the α1 domain of MICA. **(C)** Representation of the multimeric α1 peptide generated using the Multiple Antigen Peptide System (MAPS). **(D)** The α1 domain (blue, residues 1–90) forms the upper α-helix and β-sheet of the peptide-binding groove. The α2 domain (green, residues 91–182) completes the peptide-binding groove together with α1. The α3 domain (orange, residues 183–274) represents the immunoglobulin-like constant region. The transmembrane region (gray, residues 275–304) anchors MICA in the membrane, and the cytoplasmic tail (light gray, residues 305–344) is a short, positively charged intracellular extension. The peptide RDLTGNGKDLRMTLAHIKDQ (highlighted in red) corresponds to the sequence recognized by the viral particle selected during the phage display screening. **(E)** Direct ELISA assays showing reactivity of 70 scFv-phage clones against the α1 peptide in MAPS format, using BSA as a blocking agent. **(F)**. Binding of selected anti-MICA scFv clones to recombinant MICA assessed by ELISA. Data are expressed as fold-change over BSA (OD_405 MICAr/mean OD_405 BSA). Individual data points represent technical triplicates (n = 3 wells) from a single representative experiment, shown with mean ± SD. For some clones, SD values are smaller than the symbol size due to minimal intra-assay variability (CV% < 3%). ** p<0.01, analyzed by Kruskal–Wallis test. **(G)** Flow cytometry analysis of selected clones on the gastric adenocarcinoma cell line MKN-45, showing specific recognition of native MICA. Unstained cells and incubation with commercial anti-MICA antibody or anti-Hemagglutinin (HA) antibody (negative control) were included, as well as novel anti-MICA scFv-c65 + anti-HA (to detect scFv through its HA epitope). **(H)** The representative graph shows the Mean Fluorescence Intensity (MFI) for each condition in MKN-45, revealing that the selected clone (scFv-c65 + anti-HA) shows an increase in MFI compared to controls, confirming its ability to recognize native MICA on the cell surface.

To direct the selection of scFvs with affinity for the α1 region of MICA, a low- polymorphic segment encompassing residues (64–83) (**Figures 1A, B**) was chosen, which corresponds to NKG2D binding site. To efficiently expose the epitopes, the corresponding peptide was synthesized using the Multiple Antigen Peptide System (MAPS) (GenScript, Piscataway, NJ, USA) (**Figure 1C**). This system consists of a lysine core with eight branches containing identical copies of the peptide RDLTGNGKDLRMTLAHIKDQ, selected based on the crystallized structure of MICA described by Li et al. (11) (**Figures 1B, D**).

The selection of clones through panning process was conducted in ELISA plates coated with 4 µg/well of the α1 peptide (MAPS) dissolved in carbonate buffer (15 mM Na_2_CO, 35 mM NaHCO, pH 9.6). To minimize nonspecific binding, various blocking proteins were employed in successive panning rounds: 1% bovine serum albumin (BSA) in PBS in the first round, 0.05% soy proteins extract in TBS in the second, and 1% casein in the third round. Subsequently, the scFv-phages were added to the coated wells and incubated for 2 h at 37°C. After exhaustive washes with TBS-Tween (0.5–0.05%), bound phages were eluted with 100 mM glycine-HCl (pH 2.2) for 10 min at room temperature and immediately neutralized with 2 M Tris base. These eluted phages, enriched for higher affinity to the antigen, were then used to infect *E. coli* ER2738. After each panning round, or following growth of liquid cultures, the phages were precipitated from the *E. coli*-infected supernatant by adding 4% (w/v) polyethylene glycol (PEG-8000) and 3% (w/v) NaCl, keeping the mixture on ice for 30 min. The mixture was then centrifuged at 10, 000 × *g* for 10–15 min at 4°C, the supernatant was discarded, and the resulting pellet was resuspended in TBS containing 1% BSA and 0.02% sodium azide (or the relevant blocking protein), followed by filtration through a 0.22 µm filter. These preparations were stored at 4°C for subsequent use in ELISA, flow cytometry, or for initiating a new panning round. This procedure was repeated for 3–4 rounds, progressively increasing the rigor of washes and varying the blocking protein, aiming to enrich clones with high specificity and potentially increased affinity for the MICA α1 region.

Upon completion of the selection cycles, 70 clones were randomly chosen for a preliminary assessment of reactivity by direct ELISA. Each clone was cultured individually, the phages were purified using the PEG/NaCl protocol, and tested against plates coated with recombinant MICA (4 µg/mL) or the α1 peptide (MAPS) (4 µg/mL). As a control, wells were coated with BSA or soy protein. After incubating the scFv-phages (2 h, 37°C) and washing with PBS-Tween, an anti-M13 pVIII antibody conjugated to horseradish peroxidase (HRP) (1:5, 000 dilution) (GE Healthcare, USA) was added, and the reaction was developed with ABTS to measure the specific binding of each clone. Those clones exhibiting a high signal in the preliminary ELISA were amplified, and their DNA was sequenced by the Sequencing Service of the Pontificia Universidad Católica de Chile (Santiago, Chile). The selected clone (c65) was then assessed by flow cytometry to confirm their specific recognition of native MICA.

To assess recognition of native MICA, the human gastric adenocarcinoma cell line MKN-45, derived from a poorly differentiated tumor, was employed. Once they reached the appropriate confluence (approximately 3 days of culture), MKN-45 cells were collected and incubated with a commercial anti-MICA antibody (5 µg/mL) as a positive control (R&D Systems), or with scFv-c65 (10 µg/mL) together with an anti-HA as a secondary antibody (5 µg/mL) to detect the hemagglutinin tag (YPYDVPDYA) present in the scFv-c65. Flow cytometry was used to determine the mean fluorescence intensity (MFI) and the percentage of fluorescent cells, allowing relative quantification of the scFv’s specific binding to native MICA expressed on the surface of tumor cells.

### 2.6 Expression and purification of anti-MICA c65 antibody

We designed two expression vectors encoding the heavy (pOpt-aMICA) and light (pOpt-light) chains of the antibody. The constructs were synthesized by GenScript Biotech (Piscataway, NJ, USA) incorporating the optimized promoter described by Zúñiga et al. (2019) (29) and the backbone of the pOptiVEC™-TOPO^®^ vector (Invitrogen, Carlsbad, CA, USA).

Production of anti-MICA c65 antibody was carried out by transient cotransfection of ExpiCHO cells with independent plasmids for the heavy and light chains using the ExpiCHO™ Expression System Kit (Gibco™, Thermo Fisher Scientific, USA). Cells were grown in ExpiCHO expression medium in a shake flask (Techne, Thermo Fisher Scientific, USA), maintained at 37°C with 5% CO_2_ and constant shaking at 60 rpm on a MCS-104 magnetic stirrer (Techne, UK) until a cell density of 1×10^6^ cells/mL was reached. For transfection, a total of 30 µg of plasmid DNA was used in a volume of 30 mL, following the manufacturer’s instructions. Next, the incubation temperature was reduced to 32°C to improve stability and antibody yield, maintaining the culture for a period of 8–12 days, depending on cell viability. The culture supernatants were collected and centrifuged at 5, 000 × *g* for 30 min at 4°C to remove cells and debris. Subsequently, supernatants were stored at -20°C with a protease inhibitor cocktail (Millipore, Germany), until purification.

Antibody purification was performed using the ÄKTAPurifier™ system (GE Healthcare, USA) equipped with a Frac-920 collector and HiTrap™ Protein G HP purification columns (Cytiva, USA), following the manufacturers’ instructions. Culture supernatants were added to the Protein G column using 20 mM Na_3_PO_4_ binding buffer at pH 7.0. Bound antibodies were then eluted from the column with 0.1 M glycine buffer at pH 2.0 (Winkler, Chile), which disrupts antibody-Protein G binding; antibodies were immediately neutralized with 1 M Tris buffer at pH 9.0 (Winkler, Chile) to preserve antibody integrity. Antibody-enriched fractions were dialyzed with dialysis cassettes (Thermo Fisher Scientific, USA) for storage in PBS buffer at pH 7.4, following the manufacturer’s instructions.

To assess the purity and identity of purified antibodies, a Bio-Rad electrophoresis system was used. A 12% polyacrylamide gel containing 0.1% SDS was prepared, and samples with a mass of 12 µg were loaded. Separation was performed by applying a voltage of 80 V for 15 min, followed by 120 V for 90 min. The gel was further stained with Coomassie blue for 60 min and incubated in a 25% methanol solution for 3 h. The destaining procedure was repeated with fresh 25% methanol for additional 3 h. Finally, the washed gel was scanned in high resolution with an automatically optimized exposure time for improved definition using the iBright FL1500 Imaging System (Thermo Fisher Scientific, USA).

### 2.7 Antibody quantification by ELISA

ELISA plates (Thermo Fisher Scientific, USA) were coated with AffiniPure goat anti-human IgG antibody, Fcγ fragment specific (Jackson ImmunoResearch, USA) and incubated overnight at 4°C. Plate wells were blocked with a 0.1% PBS-Tween solution supplemented with 1% BSA for 2 h at 37°C. Anti-MICA c65 samples and serial dilutions of ChromPure human IgG standard (Jackson ImmunoResearch, USA) were added to the plates and incubated at 37°C for 2 h. Wells were then incubated with a peroxidase-conjugated AffiniPure goat anti-human IgG antibody, F(ab’)_2_ fragment specific (Jackson ImmunoResearch, USA) for 1 h at 37°C. The enzymatic reaction was initiated with the addition of 3, 3’, 5, 5’-tetramethylbenzidine (TMB) substrate (Thermo Fisher Scientific, USA) and stopped with 2 M sulfuric acid. The absorbance was measured at 450 nm on the Synergy™ HT Multi-Mode Microplate Reader and Gen5™ Data Analysis software (both from BioTek, USA). The data obtained were analyzed using a standard curve to determine the concentration of antibodies in the samples.

### 2.8 ELISA binding assay

ELISA plates were coated with recombinant MICA (rMICA), which include both α1 and α2 folded domains, at 3, 6 or 12 µg/mL in PBS and incubated overnight at 4°C. Plate wells were further blocked with 0.05% PBS-Tween supplemented with 1% BSA for 2 h at 37°C. Serial dilutions of anti-MICA c65 antibody in blocking solution, with concentrations ranging from 177 nM to 0.08 nM, were added and incubated for 2 h at 37°C. This concentration range was previously tested to ensure a saturated signal has been achieved in the ELISA. Next, plates were incubated, for 1 h at 37°C, with an HRP-conjugated goat polyclonal antibody (pAb) against human IgG (Abcam, UK) to detect anti-MICA c65. The enzymatic reaction was initiated by adding TMB substrate and stopped with 2 M sulfuric acid. The absorbance was measured at 450 nm on the Synergy™ HT Multi-Mode Microplate Reader and Gen5™ Data Analysis software. To determine affinity parameters, absorbance values were plotted as a function of antibody concentration to generate binding curves for each rMICA coating concentration. OD50 values, defined as the antibody concentration required to reach 50% of the maximum optical density for each binding curve, were determined using GraphPad Prism v.10 software (GraphPad Software, USA). The apparent dissociation constant (app K_d_) was calculated from pairs of OD50 values obtained at different coating concentrations (3 vs 6, 3 vs 12, and 6 vs 12 µg/mL), yielding three independent app K_d_ estimates. The reported app K_d_ value corresponds to the mean ± SD of these three pairwise calculations, following the methodology described by Beatty et al. (30).

### 2.9 Competitive ELISA assay

ELISA plates were coated with rMICA (6 or 12 µg/mL) in PBS and incubated overnight at 4°C. Subsequently, wells were blocked with 0.05% PBS-Tween supplemented with 1% BSA for 2 h at 37°C. Recombinant human NKG2D-His protein was added at a fixed concentration of 281 nM, while increasing concentrations of anti-MICA c65 antibody, ranging from 0 to 323 nM, were incubated at 37°C for 2 h. Detection of NKG2D binding was performed using an HRP-conjugated anti-His antibody (Rockland, USA) with a 1 h incubation at 37°C. The enzymatic reaction was initiated by adding TMB substrate and stopped with 2 M sulfuric acid. The absorbance was measured at 450 nm on the Synergy™ HT Multi-Mode Microplate Reader and Gen5™ Data Analysis software. The percentage binding of NKG2D was calculated relative to the maximum OD observed in wells that did not receive anti-MICA c65 antibody. The IC_50_ values were determined by fitting the data to a nonlinear regression model using GraphPad Prism software. The inhibition constant (K_i_) was calculated using the Cheng-Prusoff equation (31), considering the app K_d_ of the interaction of recombinant MICA and NKG2D at 240 nM (**Supplementary Figure S1**).

### 2.10 Antibody binding to native MICA by flow cytometry

GES-1, MKN-45 and K562 cell lines which express different MICA alleles were cultured for 3 days in RPMI-1640 medium supplemented with 10% FBS, 1% penicillin-streptomycin, and 1% L-glutamine. The MICA alleles expressed by these cell lines (*008, *009/049 and *012, respectively)* were determined in this study by MICA genotyping using the same methodology previously described (4) (**Supplementary Figure S2**), respectively. Raw genotyping data are provided in the public repository of the University of Chile. In addition, we used CHO cells stably expressing MICA*008, for testing preliminary antibody titrations to assess binding specificity and to select the optimal anti-MICA c65 working concentration for subsequent binding assays (**Supplementary Figure S3**). Cell lines were harvested, counted using a hemocytometer, and resuspended at a density of 1 × 10^6^ cells per condition. Cells were then incubated in Fc block solution (1:500 dilution) (Clone 2.4G2 SSK) for 20 min at 37°C. To assess antibody binding capacity, cells were incubated with 1 µg of anti-MICA c65 antibody for 1 h at 4°C and detected with a FITC-conjugated anti-human IgG (Fc-specific) goat secondary antibody. Controls included cells stained with commercial mouse monoclonal anti-MICA antibody conjugated to phycoerythrin (PE) (1:100 dilution) or with mouse IgG2b antibody isotype (1:100 dilution) (both from R&D Systems, USA) for 30 min at 4°C. Cells were washed with PBS-1% FBS and centrifuged at 300 × *g* for 5 min. Pellets were resuspended in 200 μL of PBS-1% FBS for immediate analysis on a BD LSRFortessa™ X-20 flow cytometer (BD Biosciences, USA). Data were analyzed with FlowJo v10 software (Tree Star Inc., USA) acquiring at least 10, 000 events per sample.

### 2.11 Antibody-dependent cell phagocytosis assays

ADCP assays were carried out using co-cultures of U937-derived macrophages (Effector, E) and GES-1 gastric epithelial cells expressing MICA protein (Target, T). U937 cells stably expressing enhanced green fluorescent protein (EGFP) (26) were differentiated into macrophages by incubation with 10 ng/mL PMA for 48 h at 37°C. GES-1 cells were labeled with the fluorescent probe TFL4 (OncoImmunin, Inc, USA) (1:2000 dilution) for 15 min in the dark and incubated for 1 h at 37°C with one of the following treatments: anti-MICA c65 antibody (10 μg), anti-CD20 antibody (10 μg) as the isotype control, or PBS as a control. After incubation with antibody, GES-1 cells were co-cultured with the differentiated U937 macrophages at an E:T ratio of 1:2 for 2 h at 37°C. Cells were then washed with PBS and centrifuged at 300 × *g* for 5 min. The cell pellet was fixed with 200 μL of 4% paraformaldehyde for 15 min in the dark. Cells were then washed and resuspended in PBS for flow cytometry analysis. Approximately 50, 000 events were acquired per condition using the BD LSR Fortessa X-20 flow cytometer. Data were analyzed using FlowJo software. For quantitative analysis, the percentage of double-positive EGFP^+^/TFL4^+^ macrophages were normalized to the PBS control at 37°C for each of the four independent experiments.

For confocal microscopy, GES-1 cells were stained with CellTracker™ Orange (Thermo Fisher Scientific, USA) before treatment. U937-derived macrophages were co-cultured with GES-1 cells at an E:T ratio of 1:2 in Lab-Tek chambered slides (Thermo Fisher Scientific, USA) and incubated under the same conditions used for ADCP. After incubation, samples were gently washed with PBS to remove non-adherent cells and fixed with 4% paraformaldehyde for 15 min in the dark. Nuclei were stained with DAPI, and preparations were mounted using DAKO mounting medium (Agilent, Denmark) to preserve fluorescence. Images were acquired directly in the Lab-Tek chambers using a Nikon C2 Plus confocal microscope (Nikon Instruments, Japan) and processed with NIS-Elements software (Nikon Instruments, Japan). Image acquisition parameters were kept constant across all samples.

### 2.12 NK activation and antibody-dependent cell cytotoxicity assays

ADCC assays were performed using K562 cells (target, T) expressing MICA and human peripheral NK cells (effector, E) purified by negative selection, as previously described. K562 cells were incubated for 30 min at 37°C with either anti-MICA c65 antibody (5 μg), anti-TNF-alpha antibody (5 μg) as an isotype, or PBS as a control. Subsequently, K562 cells were co-incubated with NK cells at a 10:1 E:T ratio for 5 h at 37°C and 5% CO_2_. After incubation, cells were stained with FITC Annexin V (Biolegend, USA) with annexin binding buffer to evaluate apoptosis. K562 target cells were identified based on forward and side scatter (FSC/SSC) parameters. Approximately 50, 000 events were recorded per condition using a BD LSR Fortessa X-20 flow cytometer, and data were analyzed using FlowJo software. Experiments were performed in three independent biological replicates.

### 2.13 Complement activation assays

Complement activation assays were performed using CHO cells transfected to express the allelic variant MICA*008 (CHO-008 cell line). Cells were incubated for 30 min at 37°C and 5% CO_2_ with either anti-MICA c65 antibody (10 µg), anti-TNF-alpha antibody (10 µg) as isotype, or PBS as a vehicle control. Subsequently, normal human serum from healthy donors was added at a final concentration of 25%, either in its active or heat-inactivated form (heated at 57°C for 40 min), and the samples were incubated for 1 h at 37°C and 5% CO_2_. After incubation, cells were washed and stained with the viability dye Zombie NIR (BioLegend, USA) for 20 min in the dark. Cells were then incubated with the primary antibody Goat anti-Human Complement C1q (Sigma-Aldrich, USA) for 30–45 min, followed by the secondary antibody donkey anti-Goat IgG (H+L), Alexa Fluor™ 488 (Thermo Fisher Scientific, USA) for an additional 30–45 min. After washing, cells were resuspended in 200 µL of PBS, and analyzed by flow cytometry (BD LSR Fortessa X-20, BD Biosciences), with approximately 50, 000 events recorded per condition. Data were analyzed using FlowJo v10 software (Tree Star, USA). Experiments were performed in three independent biological replicates.

### 2.14 Statistical analysis

Statistical analyses were performed using one-way analysis of variance (ANOVA) and Bonferroni´s *post hoc* analysis using Graph Pad Prism 10 (Version 10.3.1) software. Statistical significance was set at p < 0.05.

## 3 Results

### 3.1 Generation and selection of anti-MICA scFv clones

After completing the affinity selection through panning rounds of scFv-phages targeting the α1 peptide of MICA in MAPS format, 70 randomly chosen clones were analyzed by direct ELISA against both α1 peptide in MAPS format and recombinant MICA. Approximately 10-15% of these clones displayed binding values twice as high as those measured in wells blocked with BSA or soy protein, indicating strong specific reactivity (**Figures 1E, F**).

The clones showing the highest signals (against α1 region and recombinant MICA) were cultured individually, and sequencing revealed that clones 11, 41, and 65 shared the same nucleotide sequence (data not shown). Given their strong and reproducible reactivity in binding assays, these clones were selected for further engineering. Their sequences were subsequently included in patent US11518811B2, which supports the novelty of these fragments.

To confirm the ability of the selected scFv clones to recognize native MICA, flow cytometry assays were performed using MKN-45 cells. The scFv clones 11, 41, and 65 showed a significant increase in fluorescence compared to negative controls, confirming their binding to native MICA (**Figures 1G, H**). These findings supported the selection of the scFv clone 65 (c65) for further engineering and development into a fully human antibody format.

### 3.2 Production of a fully human antibody against MICA

**Figure 2:**
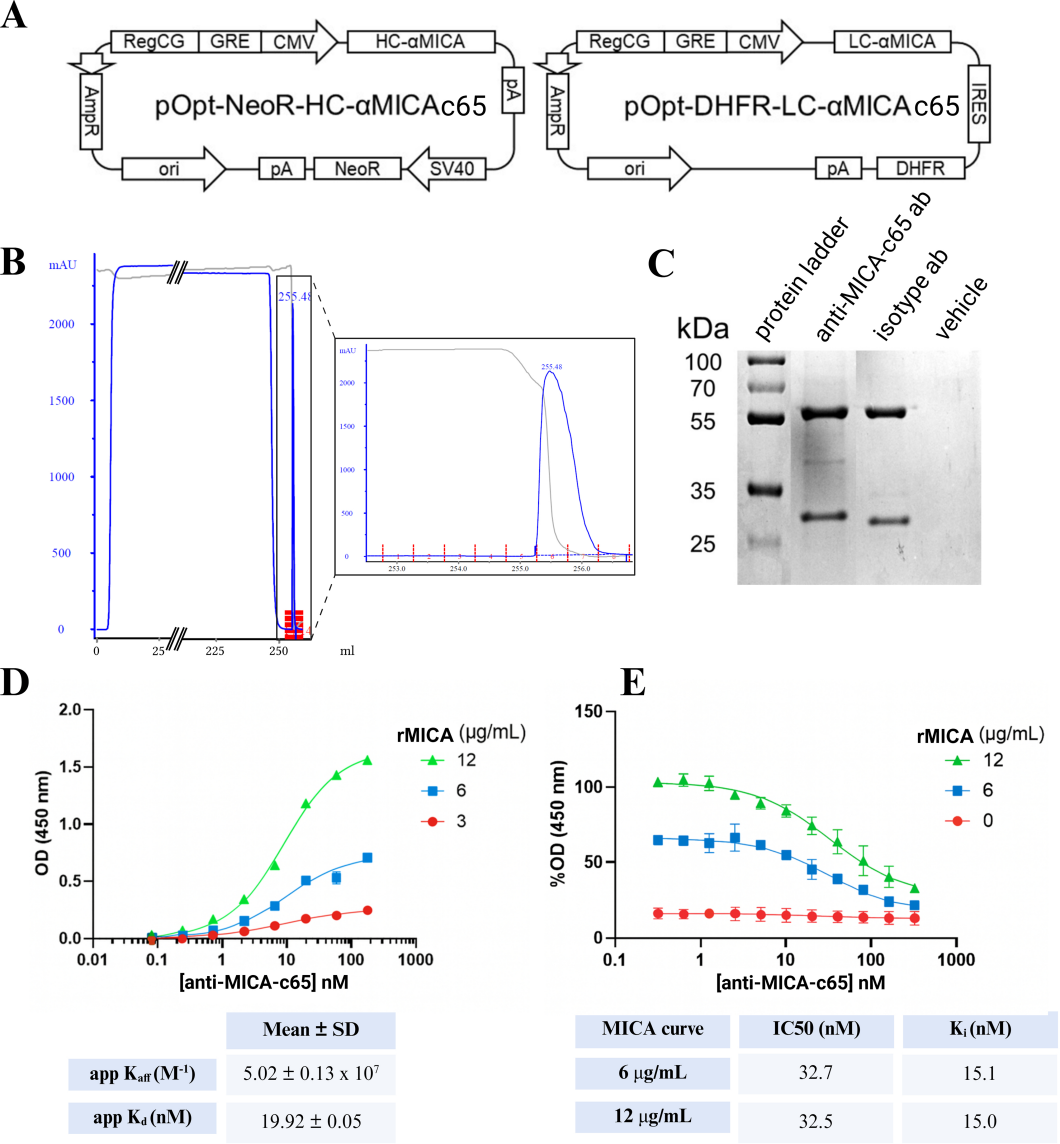
Production and biochemical characterization of the fully human anti-MICA c65 antibody. **(A)** Schematic representation of the antibody expression strategy in ExpiCHO cells using dual vectors encoding heavy and light chains. **(B)** FPLC chromatogram of Protein G-purified antibody. **(C)** SDS-PAGE under reducing conditions. Lane 1: protein ladder; Lane 2: anti-MICA c65 antibody; Lane 3: anti-CD20 antibody as isotype; Lane 4: PBS. **(D)** ELISA-based binding curve to rMICA at 3, 6, and 12 µg/mL. The binding data were used to calculate the apparent affinity constant (app K_aff_) and apparent dissociation constant (app K_d_), which are summarized in the table below the graph. **(E)** Competitive ELISA showing inhibition of NKG2D binding by anti-MICA c65 antibody. The percentage of optical density (%OD) reflects the amount of NKG2D-His bound to MICA (0, 6 and 12 µg/mL) on plates pre-coated with rMICA, following incubation with constant concentrations of NKG2D-His (9 µg/mL) and increasing concentrations of anti-MICA c65 antibody. Detection was performed using an HRP-conjugated anti-His antibody. Data represent the mean ± SD of two independent experiments.

Transient co-transfection of ExpiCHO cells with plasmids encoding the light and heavy chains resulted in successful expression of anti-MICA c65 IgG1, as depicted schematically in **Figures 2A–C**.

Antibody purification from cell culture supernatants was accomplished via Protein G affinity chromatography, yielding a single prominent elution peak (**Figure 2B**) upon decreasing the pH from neutral to acidic. This eluted fraction was immediately neutralized, dialyzed and quantified by ELISA, providing a final recovery of 1.5 mg of fully human anti-MICA c65 antibody. To confirm purity and identity, the sample was analyzed by SDS-PAGE, which showed predominant bands corresponding to the heavy (~55 kDa) and light (~29 kDa) chains characteristic of IgG1 molecules (**Figure 2C**). Minor additional bands of lower molecular weight were also observed in both the anti-MICA c65 and the commercial anti-CD20 antibody preparations, consistent with commonly reported IgG fragmentation or processing products, and therefore were not considered unexpected. Importantly, no unexpected bands were observed relative to the positive control (commercial IgG1 anti-CD20 antibody) or the negative controls (PBS buffer), indicating a high degree of purity and confirming the antibody’s expected molecular weight.

### 3.3 Anti-MICA c65 antibody binds recombinant MICA with high affinity and competes with NKG2D

To evaluate the binding properties of the anti-MICA c65 antibody, we performed an ELISA-based binding assay using recombinant MICA (as antigen) at increasing concentrations. **Figure 2D** shows a dose-dependent increase in optical density (OD) at 450 nm, confirming that the anti-MICA c65 antibody binds specifically to MICA. The aparent affinity constant (app K_aff_) was determined as 5.02 ± 0.13 × 10^7^ M, and the apparent dissociation constant (app K_d_) was calculated as 19.92 ± 0.05 nM.

To further investigate the functional activity of the anti-MICA c65 antibody, a competitive ELISA was performed to assess its ability to block the interaction between MICA and the NKG2D receptor. As shown in **Figure 2E**, increasing concentrations of the anti-MICA c65 antibody resulted in a dose-dependent reduction in NKG2D binding to MICA, expressed as a percentage of OD (%OD). This effect was more pronounced at higher MICA concentrations (12 µg/mL) compared to lower concentrations (6 µg/mL). Notably, the IC_50_ values were consistent across both conditions, with 32.7 nM for 6 µg/mL and 32.5 nM for 12 µg/mL, indicating that the blocking efficacy of the anti-MICA c65 antibody is independent of antigen density within the tested range. Using the Cheng-Prusoff equation (31), the inhibition constant (K_i_) was 15 nM, further highlighting the high inhibitory potency of the anti-MICA c65 antibody.

These results demonstrate that the anti-MICA c65 antibody effectively competes with NKG2D receptor for binding to MICA, supporting its potential as a functional blocking antibody.

### 3.4 Anti-MICA c65 antibody recognizes different MICA alleles in human cancer cells

To assess the binding potential of the anti-MICA c65 antibody to different variants of MICA protein, we used GES-1, MKN-45, and K562 cell lines, which express distinct MICA alleles: 008, 009/049, and 001, respectively (**Supplementary Figure S2**). As a preliminary specificity assessment in a controlled cellular background, we also examined anti-MICA c65 staining in CHO cells, which do not endogenously express human MICA, and in CHO cells stably transfected with MICA008. This analysis identified ≈12.1% MICA-positive cells in CHO-MICA008, above the non-transfected CHO baseline (**Supplementary Figure S3**), supporting allele-dependent recognition of surface-expressed MICA.

**Figure 3:**
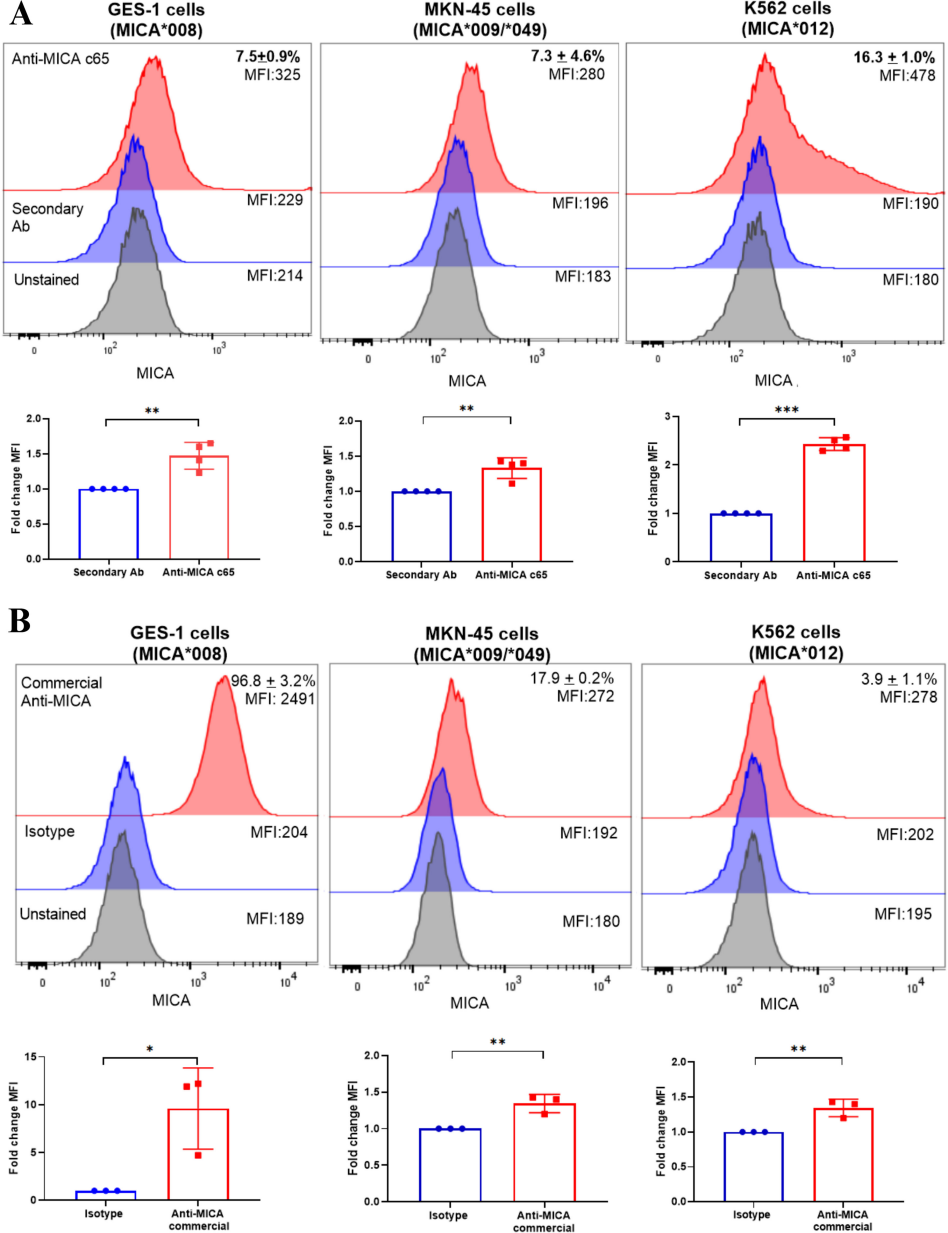
Recognition of different MICA alleles in human tumor cell lines by anti-MICA c65 antibody. **(A)** Representative histograms showing binding of the human anti-MICA c65 antibody (red) compared with the secondary antibody alone (blue) and unstained cells (grey) on GES-1, MKN-45 and K562 cells, which carry the MICA*008, *009/049 and *012 alleles, respectively. For each condition, 0.5×10^6^ cells were incubated with 1 µg anti-MICA c65 antibody and detected with a secondary FITC-conjugated anti-human IgG (Fc-specific). The histograms show MICA staining (red), secondary antibody only (blue), and unstained cells (grey), with the percentage of MICA-positive cells shown (mean ± SD). The graphs show the fold change in MFI for anti-MICA staining (with secondary antibody) relative to secondary antibody alone, from four independent experiments analyzed by Kruskal–Wallis test. Staining was performed at a single antibody concentration; fluorescence signals should therefore be interpreted qualitatively and relative to matched negative controls. **(B)** Binding of a commercial mouse monoclonal anti-MICA antibody conjugated to phycoerythrin (PE) or mouse IgG2b antibody as isotype on the same cell lines. The histograms show MICA staining (red), isotype (blue) and unstained cells (grey) in one of three experiments performed. The graphs show the fold change in MFI for the commercial anti-MICA relative to isotype antibody, from three independent experiments. Flow cytometry gating strategies for each cell line and condition to the **Supplementary Figures S4, 5**. MFI, Mean fluorescence intensity. (*): p<0.05, (**) p<0.01, (***) p<0.001.

Using an indirect staining format (anti-MICA c65 primary antibody followed by fluorescent secondary antibody), we detected MICA-positive cells in 7.5 ± 0.9% of GES-1 cells, 7.3 ± 4.6% of MKN-45 cells, and 16.3 ± 1.0% of K562 cells (**Figure 3A**; **Supplementary Figure S4**). In a separate experiment, MICA expression was also assessed with a PE-conjugated commercial murine anti-MICA antibody (direct staining), which yielded 96.8 ± 3.2% positivity in GES-1, 17.9 ± 0.2% of MKN-45, and 3.9 ± 1.1% of K562 cells (**Figure 3B**; **Supplementary Figure S5**). For MFI analysis, signals were expressed as fold change relative to the corresponding negative control (secondary antibody-only for our anti-MICA staining, and isotype control for the PE-conjugated commercial antibody). In all cases, staining produced significantly higher fold change in MFI than the respective control. Because the two antibodies were evaluated using different detection formats and fluorophores, MFI values and the percentage of positive cells are not intended for direct quantitative comparison between reagents. Nonetheless, these data support that our anti-MICA c65 antibody recognizes MICA in cells expressing multiple allelic variants.

### 3.5 Anti-MICA c65 antibody induces antibody-dependent cell phagocytosis

To determine whether the anti-MICA c65 antibody can promote macrophage-driven clearance of MICA-expressing target cells, ADCP assays were carried out using co-cultures of U937-derived macrophages and GES-1 gastric epithelial cells expressing MICA protein. GES-1 cells were labeled with the fluorescent probe TFL4 and incubated with the anti-MICA c65 antibody, a commercial anti-CD20 antibody as the isotype control, or PBS as the vehicle. Temperature controls (4°C vs 37°C) were used to distinguish active phagocytosis. Phagocytosis was evaluated by flow cytometry as the percentage of GFP^+^/TFL4^+^ macrophages (**Supplementary Figure S6**).

**Figure 4:**
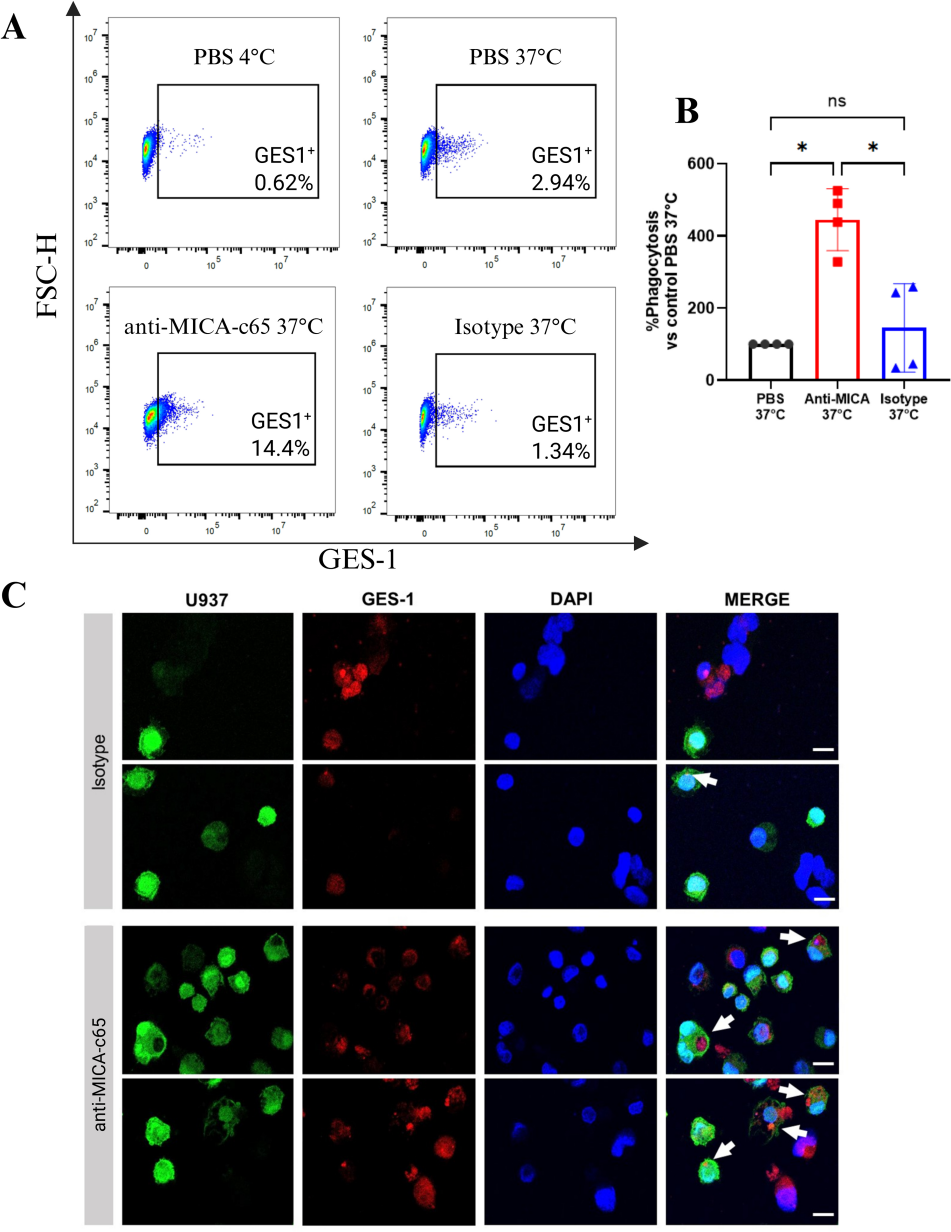
Antibody-dependent cell phagocytosis mediated by anti-MICA c65 antibody. **(A)** Dot plots showing phagocytosis by U937-EGFP macrophages. GES-1 cells labeled with the fluorescent probe TFL4 (APC) were co-cultured with U937-EGFP macrophages (FITC) in the presence of anti-MICA c65 antibody, anti-CD20 antibody as an isotype control, or vehicle (PBS) for 2 h Assays at 4 °C served as temperature controls. Dot plots illustrate the percentage of double-positive cells, corresponding to macrophages that phagocytosed GES-1 cells. **(B)** Bar graph of double-positive cell percentages. Bar graph indicates the mean ± SD of the percentage of APC+ (GES-1) cells on the FITC+ (macrophages) population, compared to 37 °C PBS control, from four independent experiments. *p<0.05, analyzed by Kruskal-Wallis test. **(C)** Confocal microscopy images of phagocytosed GES-1 cells labeled with CellTracker Orange and co-cultured with U937-EGFP macrophages (green) for 2 h in the presence of anti-MICA c65 antibody or isotype control. Two representative fields per condition are shown. White arrows highlight phagocytosed GES-1 cells. Scale bar: 10 µm.

The results showed a marked increase in the percentage of double-positive macrophages in the presence of the anti-MICA c65 antibody at 37 °C (≈14%), compared with PBS or isotype controls (≈1–3%) (**Figure 4A**), demonstrating a significant ~4-fold increase, whereas no significant difference was observed between PBS and isotype conditions (**Figure 4B**). Confocal microscopy confirmed phagocytosis of GES-1 cells by macrophages (**Figure 4C**). These results demonstrate that the anti-MICA c65 antibody effectively promotes Fc-dependent phagocytosis of MICA-expressing cells, supporting its potential to mediate tumor cell clearance by myeloid effector cells.

### 3.6 Anti-MICA c65 antibody induces antibody-dependent cell cytotoxicity

To determine whether binding of the anti-MICA c65 antibody to target cells translates into NK cell-mediated cytotoxic effector functions, we performed co-culture assays using MICA-expressing K562 target cells and primary human NK effector cells, which were isolated and characterized by flow cytometry (**Supplementary Figure S7**). Anti-MICA-mediated NK cell activation was assessed by measuring CD107a surface expression on CD56^+^CD3^−^ cells, and target cell death was evaluated by Annexin V staining. PBS and anti-TNF-α isotype antibody were included as controls.

**Figure 5:**
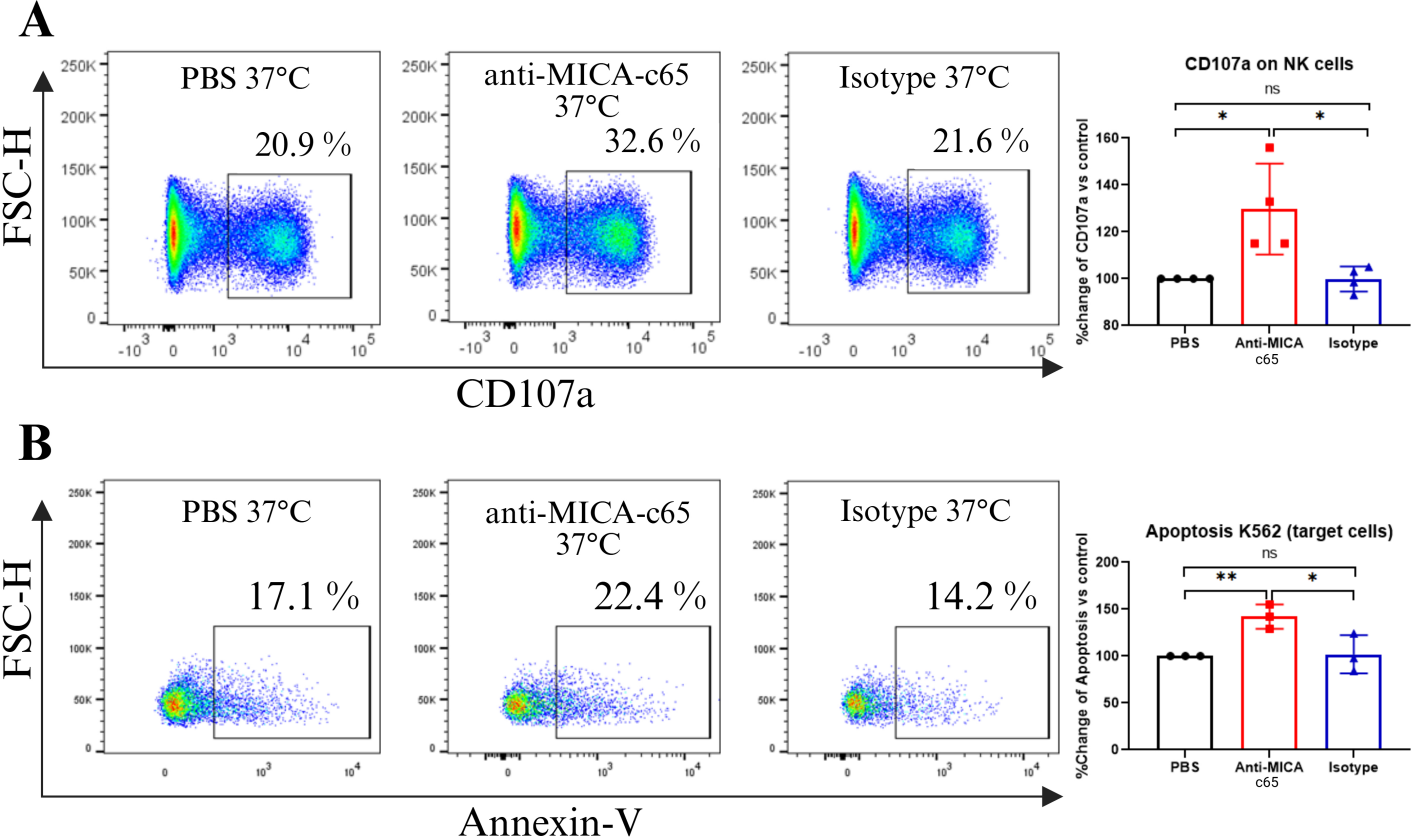
Antibody-dependent cellular cytotoxicity (ADCC) induced by anti-MICA c65 antibody. K562 target cells were incubated with anti-MICA c65 antibody and subsequently co-incubated with activated NK cells for 5 h **(A)** Dot plot of CD107a positive cells incubated with PBS (control), anti-MICA Ab (5 μg) and anti-TNF-α as an isotype control (IgG1). The bars indicate the fold change in the percentage of CD107a-positive cells compared to the control (PBS buffer). The graph shows the results of four independent experiments. (*) p<0.05, analyzed by Kruskal-Wallis test followed by the Dunn’s multiple comparisons *post hoc* test. **(B)** The percentage of apoptotic cells was assessed by Annexin V staining in the presence of Ca²^+^ under the same conditions. The bars indicate the fold change in the percentage of apoptotic K562 cells compared to the control (PBS buffer). The graph shows the results of three independent experiments. (*) p<0.05 and (**) p<0.01 analyzed by Kruskal-Wallis.

Incubation with the anti-MICA c65 antibody led to a significant increase in CD107a expression on CD56^+^CD3^−^ NK cells cells (≈32.6%) compared with PBS (≈20.9%) and isotype controls (≈21.6%) (**Figure 5A**). Additionally, the anti-MICA c65 antibody significantly increased apoptosis-mediated lysis of target cells (≈22.4%) compared with PBS (≈17.1%) and isotype controls (≈14.2%) (**Figure 5B**). These results indicate that anti-MICA c65 antibody effectively promotes NK cell activation and mediates robust ADCC against MICA-expressing target cells, supporting its potential to engage Fc-mediated effector mechanisms relevant for antitumor immune responses.

### 3.7 Anti-MICA c65 antibody triggers activation of the classical complement pathway

**Figure 6:**
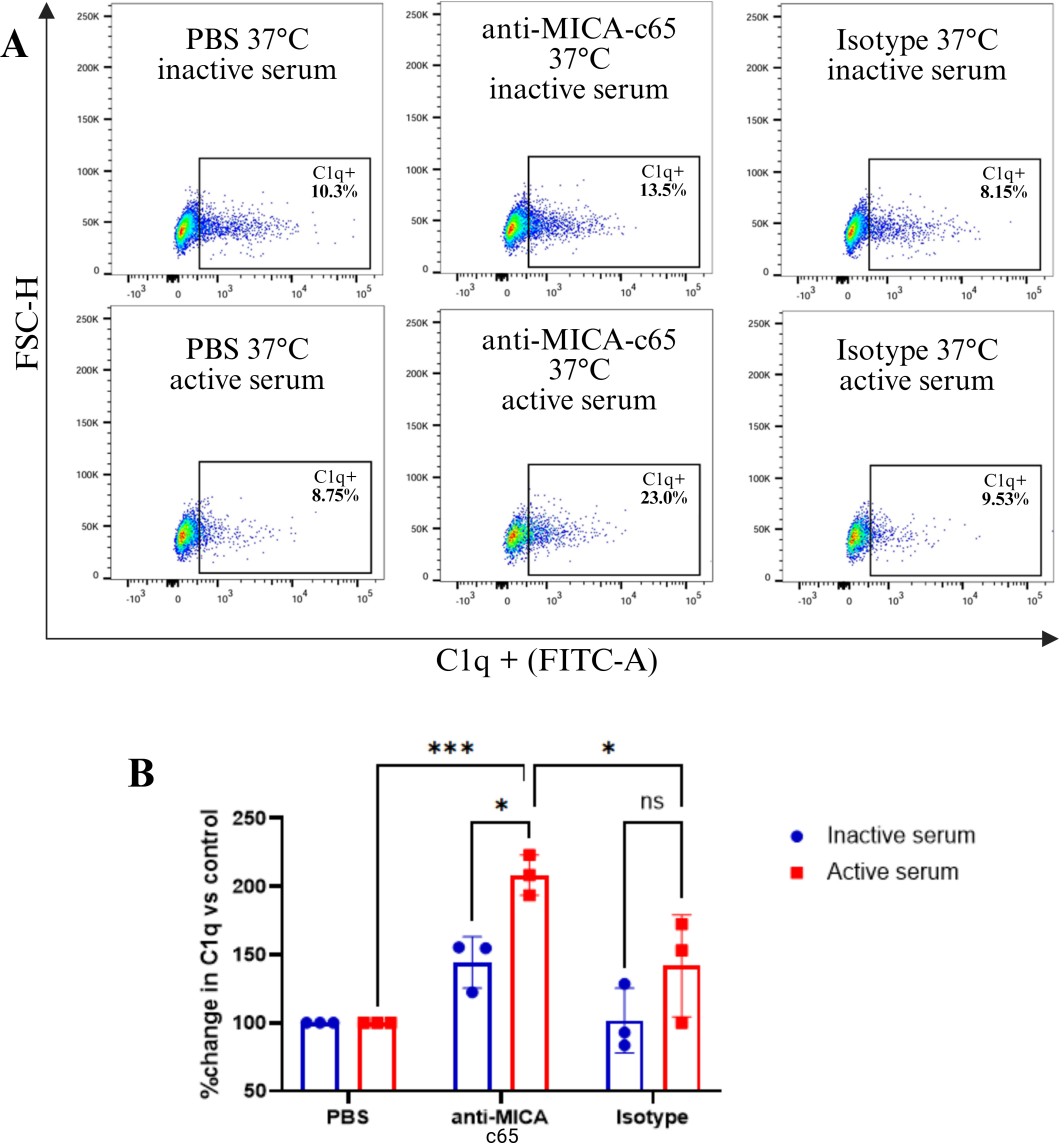
Activation of the classical complement pathway induced by anti-MICA c65 antibody. **(A)** Dot plots showing C1q binding to CHO 008 cells incubated with anti-MICA c65 antibody (10 μg), isotype antibody (10 μg), or vehicle (PBS) in the presence of either active or heat-inactivated human serum (25%). C1q detection was performed using a primary goat anti-human C1q antibody and a secondary donkey anti-goat IgG (H+L) antibody conjugated with Alexa Fluor™ 488. **(B)** Bar graph showing the percentages of C1q-positive cells. Bars represent the fold change relative to the PBS control for each individual experiment (mean ± SD, n = 3). *p* < 0.05, analyzed by Tukey’s test.

To determine whether the anti-MICA c65 antibody is capable of activating complement-mediated effector mechanisms, we evaluated the ability of the anti-MICA c65 antibody to recruit C1q. MICA-expressing CHO cells (**Supplementary Figure S3**) were treated with the anti-MICA c65 antibody, an anti-TNF-α antibody as an isotype control, or PBS as vehicle, and incubated in the presence of 25% human serum. Heat-inactivated serum served as a control for complement dependency. Flow cytometry showed that, in presence of active serum, the anti-MICA c65 treatment significantly increased the percentage of C1q+ cells (≈23%), compared with PBS and isotype controls (≈8–10%), demonstrating a 2-fold increase, whereas no significant differences were observed under heat-inactivated serum conditions (**Figures 6A, B**; **Supplementary Figure S8**). These results indicate that the anti-MICA c65 antibody efficiently recruits C1q and activates the classical complement pathway, providing an additional Fc-mediated mechanism that may contribute to its overall immunotherapeutic potential.

## 4 Discussion

MICA is a stress-inducible ligand for the activating receptor NKG2D, playing a pivotal role in antitumor immune surveillance by promoting NK and CD8^+^ T cell cytotoxicity. However, several tumor types escape this host protection mechanism through the release of MICA from the tumor cell surface, either by proteolytic shedding or via extracellular vesicles. The resulting soluble MICA binds to NKG2D on effector cells, triggering receptor internalization and downregulation of its surface expression, which in turn diminishes cytotoxic function and contributes to tumor immune suppression (17). Over the past decade, several strategies have been developed to restore MICA/NKG2D axis, particularly through antibodies targeting the α3 domain of MICA (12, 19). These antibodies inhibit proteolytic cleavage without interfering with NKG2D binding, thus preserving immune activation. Despite their promise, α3-directed antibodies do not neutralize soluble and EV-associated MICA, which continues to act as decoy molecules, sequestering NKG2D and promoting immune evasion (12).

Using a phage display strategy, we selected scFv fragments against a highly conserved linear peptide corresponding to an α-helical segment withing the MICA α1 domain. These fragments demonstrated specific recognition of native MICA expressed on human tumor cell lines as well as recombinant MICA (rMICA), with high specificity and negligible nonspecific binding in ELISA assay. Upon conversion to full-length IgG1 format, the selected clones retained their binding specificity and functional properties and displayed favorable expression levels and purity profiles in mammalian expression systems.

The ELISA binding assay revealed adequate affinity of the anti-MICA c65 antibody for its target, with app K_d_ ≈ 20 nM. Given that it was obtained by phage display technology from naïve human B-cell variable-region libraries, whose primary hits typically exhibit modest affinities (32), this provides a solid basis for subsequent affinity maturation. Affinity can be improved through computational approaches; for instance, Matthies and cols (33) reported approximately 3-fold gains of affinity for an scFv. Through this approach, it would be possible to achieve affinities comparable to those of clinically approved therapeutic antibodies, which typically range from the low nanomolar to picomolar levels (34, 35).

The observed binding characteristics described in this work suggest that the anti-MICA c65 antibody possesses the molecular features necessary for effective therapeutic application. Notably, the app K_d_ of the anti-MICA c65 antibody is lower than the reported K_d_ for the NKG2D-MICA interaction, measured at approximately 1 µM by SPR (11), as well as the experimentally determined (240 nM) by ELISA in this study. This difference highlights a competitive advantage of our antibody in binding to MICA, supporting its capacity to displace NKG2D under competitive conditions.

The relatively moderate binding affinity observed for the anti-MICA c65 antibody is consistent with its origin from a naïve human scFv library, as initial binders derived from such libraries typically display affinities in the nanomolar to micromolar range prior to affinity maturation (32). In addition, the panning strategy employed a linear peptide corresponding to an α-helical segment of the MICA α1 domain, which may not fully recapitulate the native conformational epitope. Consequently, some residues critical for antibody binding in the native MICA structure could be partially buried within the α1-α2 platform, limiting initial binding strength.

Importantly, despite this moderate affinity, biodistribution experiments in immunodeficient NOD/SCID mice bearing human MKN-45 gastric tumors demonstrated selective accumulation of the anti-MICA scFv-c65 at the tumor site compared to a control protein (**Supplementary Figure S9**). These biodistribution experiments were performed using the scFv-c65 format rather than the full-length IgG, representing a conservative assessment of tumor targeting, as scFv-c65 fragments lack Fc-mediated avidity and typically display shorter serum half-life. These findings indicate that the antibody retains sufficient *in vivo* target recognition to localize to MICA-expressing tumors, supporting its biological relevance and further development.

Together, these observations support affinity maturation as a rational next step to enhance binding strength while preserving epitope specificity, with the potential to further improve tumor targeting and therapeutic efficacy.

Consistent with this framework, the competitive ELISA results highlight the functional efficacy of the anti-MICA c65 antibody as an inhibitor of the MICA-NKG2D interaction (Ki of approximately 15 nM). This constant reflects the concentration of antibody required to inhibit NKG2D binding to MICA under equilibrium conditions and should be interpreted as a functional measure of blocking potency rather than an affinity parameter. The consistent IC_50_ values obtained here suggest that the antibody’s inhibitory capacity is largely independent of antigen density. These findings propose that the anti-MICA c65 antibody demonstrates inhibitory capabilities within the range required for effective therapeutic modulation of immune interactions. It is important to note that NKG2D blockade is not a general feature of anti-MICA antibodies. Most previously described research and therapeutic antibodies target the α3 domain and are designed to preserve MICA-NKG2D interactions while preventing ligand shedding. Therefore, direct comparisons of NKG2D inhibition across antibodies recognizing distinct MICA domains are not mechanistically informative.

By targeting a relatively conserved epitope within the α1 domain, the interface engaged by NKG2D, our antibody recognizes several prevalent MICA alleles (MICA*008, *009/049, and *012), thereby helping to mitigate challenges associated with MICA polymorphism. This population-compatible breadth supports its potential for therapeutic deployment and may also enable diagnostic applications, including epitope-resolved multiplex assays or rational antibody panels to refine MICA profiling. Notably, the relatively weak staining observed in some cellular contexts may reflect several non-mutually exclusive factors, including limited surface density of MICA, epitope accessibility on the native α1-α2 platform, and/or sequestration of antibody by soluble or shed MICA present in the culture supernatant, rather than a lack of allele recognition per se. Mapping allele-specific reactivity could, in turn, contribute to patient stratification and inform personalized MICA-directed immunotherapy.

A limitation of the present study is that anti-MICA c65 specificity at the level of flow cytometry staining was not validated using genetic approaches (e.g., siRNA-mediated knockdown or knockout of MICA) in the human cell lines analyzed. Accordingly, the flow cytometry experiments were primarily intended to demonstrate qualitative recognition of native, surface-expressed MICA rather than to provide a quantitative affinity estimate. To strengthen specificity assessment in a controlled cellular background, we included preliminary data using CHO parental cells (MICA-negative) and CHO cells stably expressing MICA*008, in which anti-MICA c65 staining was increased in CHO–MICA008 relative to the parental control. We also performed preliminary antibody titrations in CHO–MICA008 to define a working concentration that provided an adequate signal-to-background ratio for subsequent binding assays.

Functionally, the antibody mediated multiple Fc-dependent effector mechanisms, as it induced ADCC on NK cells, promoted ADCP by macrophages, and triggered classical complement pathway activation on MICA-positive cells. These mechanisms are critical for effective tumor clearance (36) and are consistent with the activity profiles of other IgG1 therapeutics, such as mAb RDM028 targeting MICA α3 domain in colorectal cancer (37) or the bispecific fusion antibody mAb04-MICA acting on VEGFR2 and MICA (38).

Beyond its direct role in tumor cell clearance, antibody-induced phagocytosis could contribute to the activation of a more robust adaptive immune response. As shown in **Figure 4**, the internalization of tumor cells by macrophages suggests a potential mechanism of cross-presentation of tumor antigens, which could favor the activation of tumor-specific naive CD8+ T lymphocytes (39, 40) and local reactivation of memory CD8+T lymphocytes (39). The combination of immediate Fc-mediated effector functions with the potential induction and/or reactivation of adaptive immunity positions this antibody as a promising component of integrated immunotherapeutic approaches, where it could complement existing treatment rather than act as a standalone agent.

In primary NK cells, the anti-MICA c65 antibody elicited significant ADCC, evidenced by increased surface CD107a, a validated marker of degranulation, and concomitant apoptosis of opsonized target cells (41). Mechanistically, NK-cell cytotoxicity is mediated by perforin/granzyme release and death-receptor signaling; thus, enhanced degranulation is consistent with increased target-cell apoptosis. The magnitude of ADCC depends on the density of CD16a (FcγRIIIa) and the Fc–receptor affinity; notably, the FCGR3A V158F polymorphism modulates IgG1 binding and has been associated with inter-individual differences in ADCC and clinical responses (42, 43). Moreover, Fc engineering that strengthens CD16a engagement, such as Fc core afucosylation, can further potentiate ADCC (44). Accordingly, genotyping *FCGR3A* V158F and other FcγR polymorphisms should be considered in future studies, especially those involving antibodies, as these variants can influence Fc–CD16a affinity and, consequently, ADCC potency.

To explore the contribution of complement, we demonstrated that the anti-MICA c65 antibody recruits C1q on MICA-expressing CHO cells in the presence of active human serum (**Figures 6A, B**), which is consistent with initiation of the classical complement pathway. According to the literature, efficient C1q engagement by IgG requires at least two Fc regions in close enough proximity on the target cell membrane, which is is determined by the antibody isotype, antigen density on the cell surface, the recognized epitope, and antigen mobility (45). In this context, despite the shedding of soluble MICA and the release of EV-associated MICA by the CHO-MICA008 line (data no shown), these cells still express MICA on the membrane (see **Supplementary Figure S3**), which is available for antibody binding to promote Fc clustering and subsequent recruitment of C1q.

Nevertheless, the cytolytic outcome does not depend exclusively on these antigen–antibody interactions. Several tumor cell types express membrane-associated complement regulators (CD46, CD55, and CD59) and may even release soluble complement inhibitors (C1-inhibitor, factor H, sCD59), thereby attenuating C4b/C3b deposition and membrane-attack-complex formation (46, 47). Our study was limited to determining a proximal readout (C1q recruitment) and did not assess distal markers of the cascade activation (C4b/C3b deposition, C5b-9) or target cell lysis. In this sense, more studies are necessary to provide evidence for effective complement-dependent cytotoxicity mediated by our antibody.

Taken together, the C1q recruitment we document in this report aligns with established determinants of classical pathway activation and suggests that, in settings with high MICA surface expression and favorable complement regulation, the anti-MICA c65 antibody could provide an additional effector mechanism (CDC) in addition to the mechanisms already demonstrated (competitive inhibition of the MICA-NKG2D interaction *in vitro* and Fc-dependent immune effector activation).

In the evolving landscape of MICA-targeted therapies, multiple strategies have been proposed. CLN-619, a humanized IgG1 in phase I (NCT05117476), binds the α3 domain of MICA/B to inhibit proteolytic shedding, thereby increasing surface ligand density while preserving NKG2D engagement and enabling Fc-mediated effector functions. In preclinical model, this approach has also been shown to reduce soluble MICA/B levels rather than directly neutralizing circulating sMICA. Similarly, DM919, developed by D2M Biotherapeutics and also in phase I trials (NCT06328673), aims to restore MICA/B surface expression and NK cell function. Prior work with BLS-MICA vaccine-like construct reported by Torres et al. (48), as well as bispecific formats (e.g., mAb04-MICA) (38) underscores the value of restoring NKG2D activity in antitumor immunity. To our knowledge, no published monospecific antibody directly blocks the NKG2D-binding site while simultaneously delivering a similarly broad Fc-effector profile.

Taken together, our results suggest that α1-directed antibodies offer unique advantages, particularly in tumors with high sMICA levels, including gastric and hematologic malignancies (34, 45), and reduced NKG2D signaling. Unlike α3-based strategies (7C6 (19, 24, 49), AHA-1031 (23), AHA-1032 antibodies), which depend on retaining surface MICA for immune activation, our approach blocks the MICA-NKG2D interactions *in vitro* while promoting direct tumor cell elimination via Fc-mediated effector functions. Although our antibody effectively blocks the MICA–NKG2D interaction *in vitro* and mediates Fc-dependent effector functions against MICA-expressing cells, the present study did not directly assess the impact of soluble MICA on antibody binding or function. As high levels of circulating sMICA could potentially compete with cell-surface MICA for antibody binding, future studies will be required to evaluate antibody performance in the presence of soluble MICA under physiologically relevant conditions. Given that MICA/NKG2D axis disruption has been associated with increased PD-L1 expression (50, 51), it is reasonable to hypothesize that α1-targeting antibodies could enhance the efficacy of immune checkpoint inhibitors, increasing CD8^+^ T cell expansion and reducing immunotherapy-associated toxicity (52). These data support a potential role for our antibody in combinatorial immunotherapy strategies, including NK cell–based strategies augmented with cytokines such IL-15, IL-18, and IL-37 (53).

The present study acknowledges several limitations that should be considered in future research. One limitation is the paucity of data regarding MICA associated with extracellular vesicles (EV-MICA), a form that contributes to immune suppression in various tumors. A critical next step will be to assess the antibody’s capacity to recognize and clear EV-MICA, thereby elucidating its potential to neutralize these vesicle-associated ligands within the tumor microenvironment. In the future, *in vivo* experimentation employing xenograft or syngeneic models is imperative to substantiate antitumor activity, pharmacodynamics, and safety.

## 5 Conclusion

In summary, we report on the development and initial characterization of a fully human IgG1 antibody directed against the α1 domain of MICA. The antibody display detectable but relatively low binding affinity, recognized a set of MICA alleles expressed on the cell types tested (including MICA*008, *009 and *012), which are frequently observed in human tumors, exhibits NKG2D-competitive binding, and mediates Fc-dependent immune effector functions. By targeting the α1 domain, this antibody provides a proof of concept for a novel MICA-based immunotherapies approach. Future studies will focus on improving the affinity, preclinical evaluation in animal models, and assessment of combinatorial potential with immune checkpoint blockade.

## Data Availability

The data presented in the study are deposited in the ‘Repositorio de datos de investigación de la Universidad de Chile’, available at https://doi.org/10.34691/UCHILE/0WXTAH.
