# Supplementary material for: A fully human IgG1 antibody targeting MICA α1 domain inhibits interaction with NKG2D and activates immune effector functions against MICA-expressing cells
Source: Front Immunol. 2026 Feb 11;17:1740184. doi: 10.3389/fimmu.2026.1740184 (PMC12932519; doi:10.3389/fimmu.2026.1740184)
Supplement: Supplementary file 1 [file DataSheet1.pdf]

Supplementary Materials

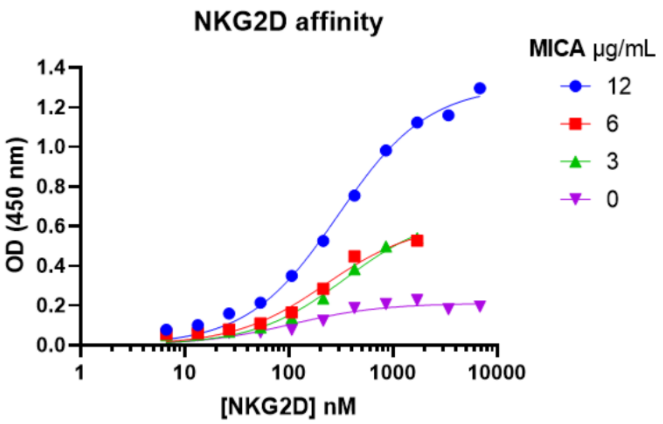

Supplementary Figure S1: ELISA determination of MICA–NKG2D binding affinity. ELISA plates were coated with recombinant MICA (rMICA) at 3, 6, or 12 µg/mL. A titration of soluble NKG2D-His (0.21–218.5 µg/mL) was added and bound receptor was detected with an HRP-conjugated anti-His antibody. Binding curves were fit by nonlinear regression to estimate the apparent affinity constant (app K_{aff}) and apparent dissociation constant (app K_d); summary values are reported in the table below the graph. Data are presented as mean ± SD from two independent experiments. App K_d= 240 nM.

```
cDNA          1
              |                                                                                           Exon 2
MICA*001      ATG GGG CTG GGC CCG GTC TTC CTG CTT CTG GCT GGC ATC TTC CCT TTT GCA CCT CCG GGA GCT GCT GCT|GAG CCC
MICA*008      --- --- --- --- --- --- --- --- --- --- --- --- --- --- --- --- --- --- --- --- --- --- --- --- ---
MICA*009      --- --- --- --- --- --- --- --- --- --- --- --- --- --- --- --- --- --- --- --- --- --- --- --- ---
MICA*012      --- --- --- --- --- --- --- --- --- --- --- --- --- --- --- --- --- --- --- --- --- --- --- --- ---
              76
              |
MICA*001      CAC AGT CTT CGT TAT AAC CTC ACG GTG CTG TCC TGG GAT GGA TCT GTG CAG TCA GGG TTT CTC ACT GAG GTA CAT
MICA*008      --- --- --- --- --- --- --- --- --- --- --- --- --- --- --- --- --- --- --- --- --T G-- --- --- ---
MICA*009      --- --- --- --- --- --- --- --- --- --- --- --- --- --- --- --- --- --- --- --- --T G-- --- --- ---
MICA*012      --- --- --- --- --- --- --- --- --- --- --- --- --- --- --- --- --- --- --- --- --- --- --- --- ---
              151
              |
MICA*001      CTG GAT GGT CAG CCC TTC CTG CGC TGT GAC AGG CAG AAA TGC AGG GCA AAG CCC CAG GGA CAG TGG GCA GAA GAT
MICA*008      --- --- --- --- --- --- --- --- -A- --- --- --- --- --- --- --- --- --- --- --- --- --- --- --- ---
MICA*009      --- --- --- --- --- --- --- --- -A- --- --- --- --- --- --- --- --- --- --- --- --- --- --- --- ---
MICA*012      --- --- --- --- --- --- --- --- --- --- --- --- --- --- --- --- --- --- --- --- --- --- --- --- ---
              226
              |
MICA*001      GTC CTG GGA AAT AAG ACA TGG GAC AGA GAG ACC AGA GAC TTG ACA GGG AAC GGA AAG GAC CTC AGG ATG ACC CTG
MICA*008      --- --- --- --- --- --- --- --- --- --- --- --G --- --- --- --- --- --- --- --- --- --- --- --- ---
MICA*009      --- --- --- --- --- --- --- --- --- --- --- --G --- --- --- --- --- --- --- --- --- --- --- --- ---
MICA*012      --- --- --- --- --- --- --- --- --- --- --- --- --- --- --- --- --- --- --- --- --- --- --- --- ---
              301
              |                                 Exon 3
MICA*001      GCT CAT ATC AAG GAC CAG AAA GAA G|GC TTG CAT TCC CTC CAG GAG ATT AGG GTC TGT GAG ATC CAT GAA GAC AAC
MICA*008      --- --- --- --- --- --- --- --- -|-- --- --- --- --- --- --- --- --- --- --- --- --- --- --- --- ---
MICA*009      --- --- --- --- --- --- --- --- -|-- --- --- --- --- --- --- --- --- --- --- --- --- --- --- --- ---
MICA*012      --- --- --- --- --- --- --- --- -|-- --- --- --- --- --- --- --- --- --- --- --- --- --- --- --- ---
              376
              |
MICA*001      AGC ACC AGG AGC TCC CAG CAT TTC TAC TAC GAT GGG GAG CTC TTC CTC TCC CAA AAC CTG GAG ACT AAG GAA TGG
MICA*008      --- --- --- --- --- --- --- --- --- --- --- --- --- --- --- --- --- --- --- --- --- --- G-- --- ---
MICA*009      --- --- --- --- --- --- --- --- --- --- --- --- --- --- --- --- --- --- --- G-- --- --- G-- --- ---
MICA*012      --- --- --- --- --- --- --- --- --- --- --- --- --- --- --- --- --- --- --- --- --- --- G-- --- ---
              451
              |
MICA*001      ACA ATG CCC CAG TCC TCC AGA GCT CAG ACC TTG GCC ATG AAC GTC AGG AAT TTC TTG AAG GAA GAT GCC ATG AAG
MICA*008      --- G-- --- --- --- --- --- --- --- --- --- --- --- --- --- --- --- --- --- --- --- --- --- --- ---
MICA*009      --- G-- --- --- --- --- --- --- --- --- --- --- --- --- --- --- --- --- --- --- --- --- --- --- ---
MICA*012      --- --- --- --- --- --- --- --- --- --- --- --- --- --- --- --- --- --- --- --- --- --- --- --- ---
              526
              |
MICA*001      ACC AAG ACA CAC TAT CAC GCT ATG CAT GCA GAC TGC CTG CAG GAA CTA CGG CGA TAT CTA AAA TCC GGC GTA GTC
MICA*008      --- --- --- --- --- --- --- --- --- --- --- --- --- --- --- --- --- --- --- --- G-- --- --- --- ---
MICA*009      --- --- --- --- --- --- --- --- --- --- --- --- --- --- --- --- --- --- --- --- G-- --- A-- --- ---
```

```
MICA*012      --- --- --- -T- --- --- --- --- --- --- --- --- --- --- --- --- --- --- --- --- --- --- --- --- ---
              601
              |                 Exon 4
MICA*001      CTG AGG AGA ACA G|TG CCC CCC ATG GTG AAT GTC ACC CGC AGC GAG GCC TCA GAG GGC AAC ATT ACC GTG ACA TGC
MICA*008      --- --- --- --- -|-- --- --- --- --- --- --- --- --- --- --- --- --- --- --- --- --C --- --- --- ---
MICA*009      --- --- --- --- -|-- --- --- --- --- --- --- --- --- --- --- --- --- --- --- --- --C --- --- --- ---
MICA*012      --- --- --- --- -|-- --- --- --- --- --- --- --- --- --- --- --- --- --- --- --- --- --- --- --- ---
              676
              |
MICA*001      AGG GCT TCT GGC TTC TAT CCC TGG AAT ATC ACA CTG AGC TGG CGT CAG GAT GGG GTA TCT TTG AGC CAC GAC ACC
MICA*008      --- --- --C A-- --- --- --- C-- --- --- -T- --- -C- --- --- --- --- --- --- --- --- --- --- --- ---
MICA*009      --- --- --C A-- --- --- --- C-- --- --- --- --- -C- --- --- --- --- --- --- --- --- --- --- --- ---
MICA*012      --- --- --- --- --- --- --- --- --- --- --- --- --- --- --- --- --- --- --- --- --- --- --- --- ---
              751
              |
MICA*001      CAG CAG TGG GGG GAT GTC CTG CCT GAT GGG AAT GGA ACC TAC CAG ACC TGG GTG GCC ACC AGG ATT TGC CAA GGA
MICA*008      --- --- --- --- --- --- --- --- --- --- --- --- --- --- --- --- --- --- --- --- --- --- --- -G- ---
MICA*009      --- --- --- --- --- --- --- --- --- --- --- --- --- --- --- --- --- --- --- --- --- --- --- --- ---
MICA*012      --- --- --- --- --- --- --- --- --- --- --- --- --- --- --- --- --- --- --- --- --- --- --- --- ---
              826
              |                                                                                   Exon 5
MICA*001      GAG GAG CAG AGG TTC ACC TGC TAC ATG GAA CAC AGC GGG AAT CAC AGC ACT CAC CCT GTG CCC TCT G|GG AAA GTG
MICA*008      --- --- --- --- --- --- --- --- --- --- --- --- --- --- --- --- --- --- --- --- --- --- -|-- --- ---
MICA*009      --- --- --- --- --- --- --- --- --- --- --- --- --- --- --- --- --- --- --- --- --- --- -|-- --- ---
MICA*012      --- --- --- --- --- --- --- --- --- --- --- --- --- --- --- --- --- --- --- --- --- --- -|-- --- ---
              901
              |
MICA*001      CTG GTG CTT CAG AGT CAT TGG CAG ACA TTC CAT GTT TCT GCT GTT GCT GCT G.CT GCT ..... ..... ..... ...ATT TTT GTT
MICA*008      --- --- --- --- --- --- --- --- --- --- --- --- --- --- --- --- --- -G-- --- GCT.. ..... ..... ...--- --- ---
MICA*009      --- --- --- --- --- --- --- --- --- --- --- --- --- --- --- --- --- -.-- --- GCTGC T.... ..... ...--- --- ---
MICA*012      --- --- --- --- --- --- --- --- --- --- --- --- --- --- --- --- --- -.-- --- ..... ..... ..... ...--- --- ---
              967
              |
MICA*001      ATT ATT ATT TTC TAT GTC CGT TGT TGT AAG AAG AAA ACA TCA GCT GCA GAG GGT CCA GA|
MICA*008      --- --- --- --- --- --- --- --- --- --- --- --- --- --- --- --- --- --- --- --|
MICA*009      --- --- --- --- --- --- --- --- --- --- --- --- --- --- --- --- --- --- --- --|
MICA*012      --- --- --- --- --- --- --- --- --- --- --- --- --- --- --- --- --- --- --- --|
```

Supplementary Figure S2: Genotyping of *MICA* alleles in human cell lines GES-1, MKN-45, and K562. Alignment of the coding sequences (cDNA) of the MICA gene obtained from cell lines, compared to the reference sequence of the *MICA001* allele. The exons are represented in different colors for easy identification: blue (exon 2), red (exon 3), green (exon 4), and gray (exon 5). Alignment analysis reveals specific nucleotide variations that allow the identification of the allele expressed by each cell line. In particular, GES-1 cells show substitutions compatible with the *MICA*008* allele, MKN-45 cells with the *MICA*009* allele, and K562 cells with the *MICA*012* allele. These differences are mainly concentrated in exons 3 and 4, where the polymorphisms that distinguish the different alleles of the gene are found. Likewise, in exon 5, short tandem repeats (STRs) formed by the GCT triplet, which encode alanine residues, stand out.

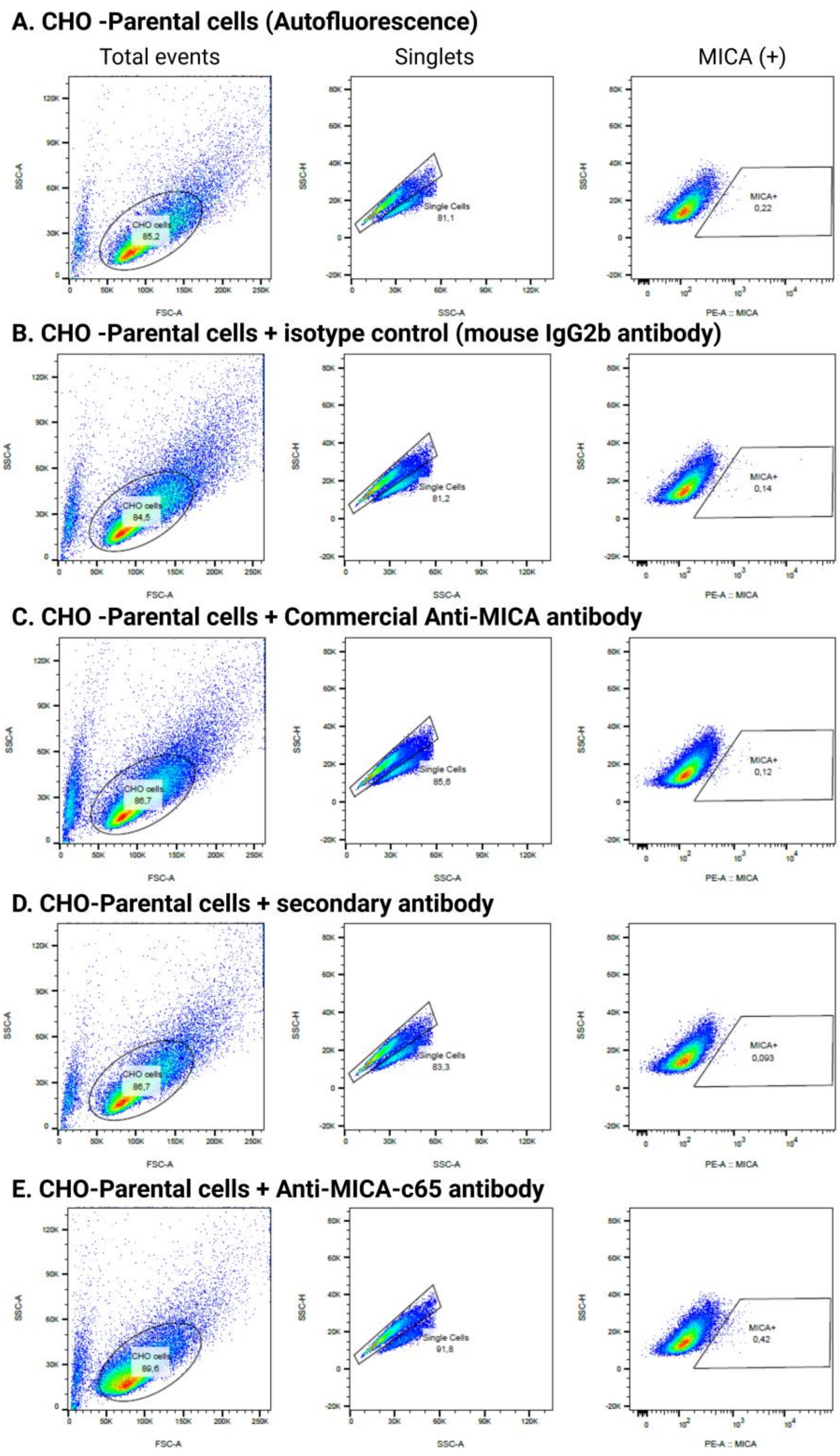
A. CHO -Parental cells (Autofluorescence)
Total events
Singlets
MICA (+)
CHO cells 85,2
Single Cells 81,1
MICA+ 0,22
B. CHO -Parental cells + isotype control (mouse IgG2b antibody)
CHO cells 84,5
Single Cells 81,2
MICA+ 0,14
C. CHO -Parental cells + Commercial Anti-MICA antibody
CHO cells 86,7
Single Cells 85,6
MICA+ 0,12
D. CHO-Parental cells + secondary antibody
CHO cells 86,7
Single Cells 83,3
MICA+ 0,093
E. CHO-Parental cells + Anti-MICA-c65 antibody
CHO cells 89,6
Single Cells 91,8
MICA+ 0,42
SSC-A
FSC-A
SSC-H
PE-A :: MICA

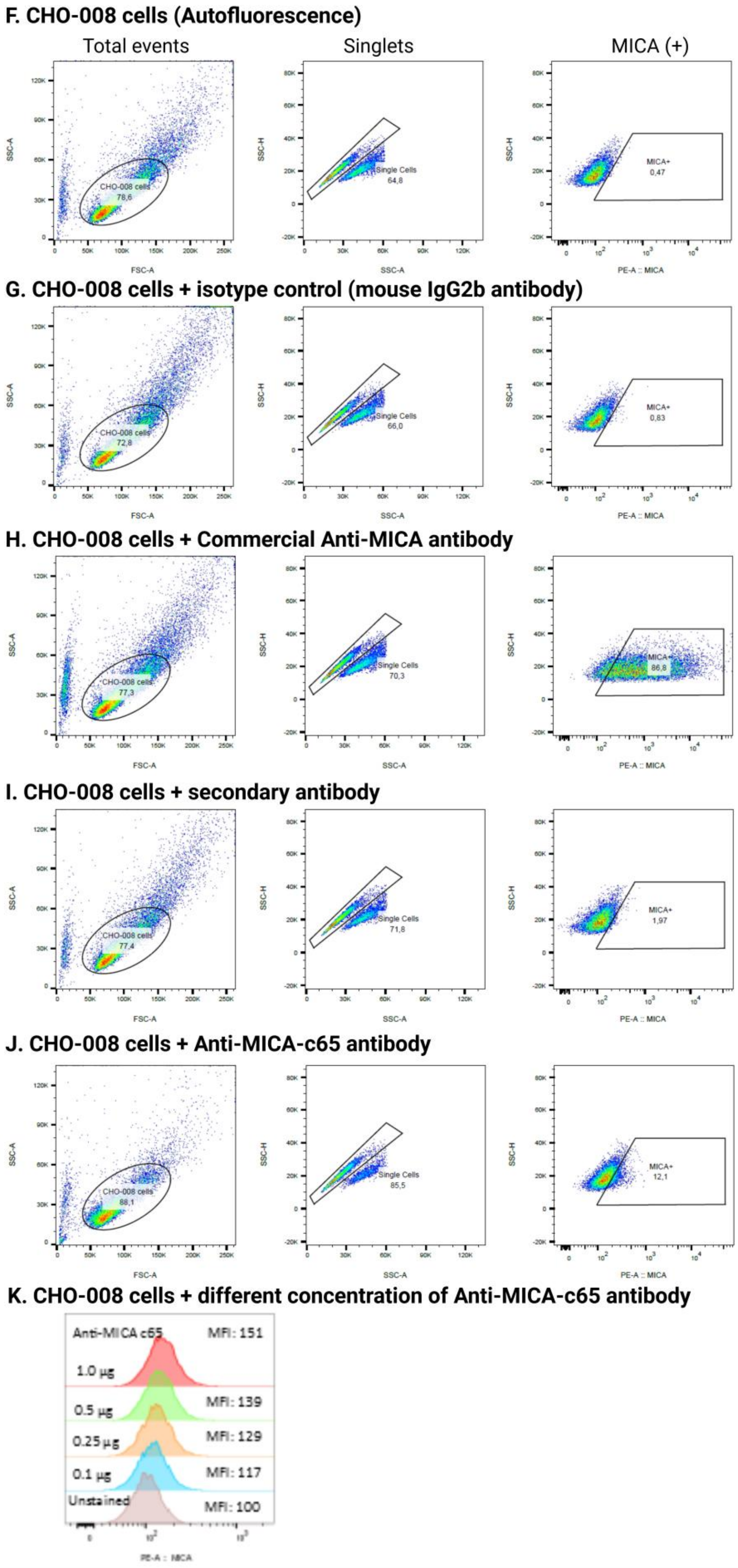
F. CHO-008 cells (Autofluorescence)
Total events
Singlets
MICA (+)
CHO-008 cells 78,6
Single Cells 64,8
MICA+ 0,47
G. CHO-008 cells + isotype control (mouse IgG2b antibody)
CHO-008 cells 72,8
Single Cells 66,0
MICA+ 0,83
H. CHO-008 cells + Commercial Anti-MICA antibody
CHO-008 cells 77,3
Single Cells 70,3
MICA+ 86,8
I. CHO-008 cells + secondary antibody
CHO-008 cells 77,4
Single Cells 71,8
MICA+ 1,97
J. CHO-008 cells + Anti-MICA-c65 antibody
CHO-008 cells 88,1
Single Cells 85,5
MICA+ 12,1
SSC-A
FSC-A
SSC-H
PE-A :: MICA
K. CHO-008 cells + different concentration of Anti-MICA-c65 antibody
Anti-MICA c65
MFI: 151
1.0 µg
0.5 µg
MFI: 139
0.25 µg
MFI: 129
0.1 µg
MFI: 117
Unstained
MFI: 100
PE-A :: MICA

Supplementary Figure S3. Gating strategy for CHO parental and CHO-MICA*008 cells and flow-cytometry binding of anti-MICA c65 and a commercial anti-MICA antibody. (A, F) Unstained cells (autofluorescence). (B, G) Isotype control (mouse IgG2b) conjugated to phycoerythrin (PE). (C, H) Commercial mouse monoclonal anti-MICA antibody conjugated to PE. (D, I) Secondary antibody only (FITC-conjugated goat anti-human IgG [Fc-specific]). (E, J) Anti-MICA c65 antibody (1 μg). (K) Anti-MICA c65 titration at 0.1, 0.25, 0.5, and 1 μg. For all conditions, 0.5×10^6 cells were incubated at 4°C for 1 h as described in the Method section. Data are shown from a single independent experiment.

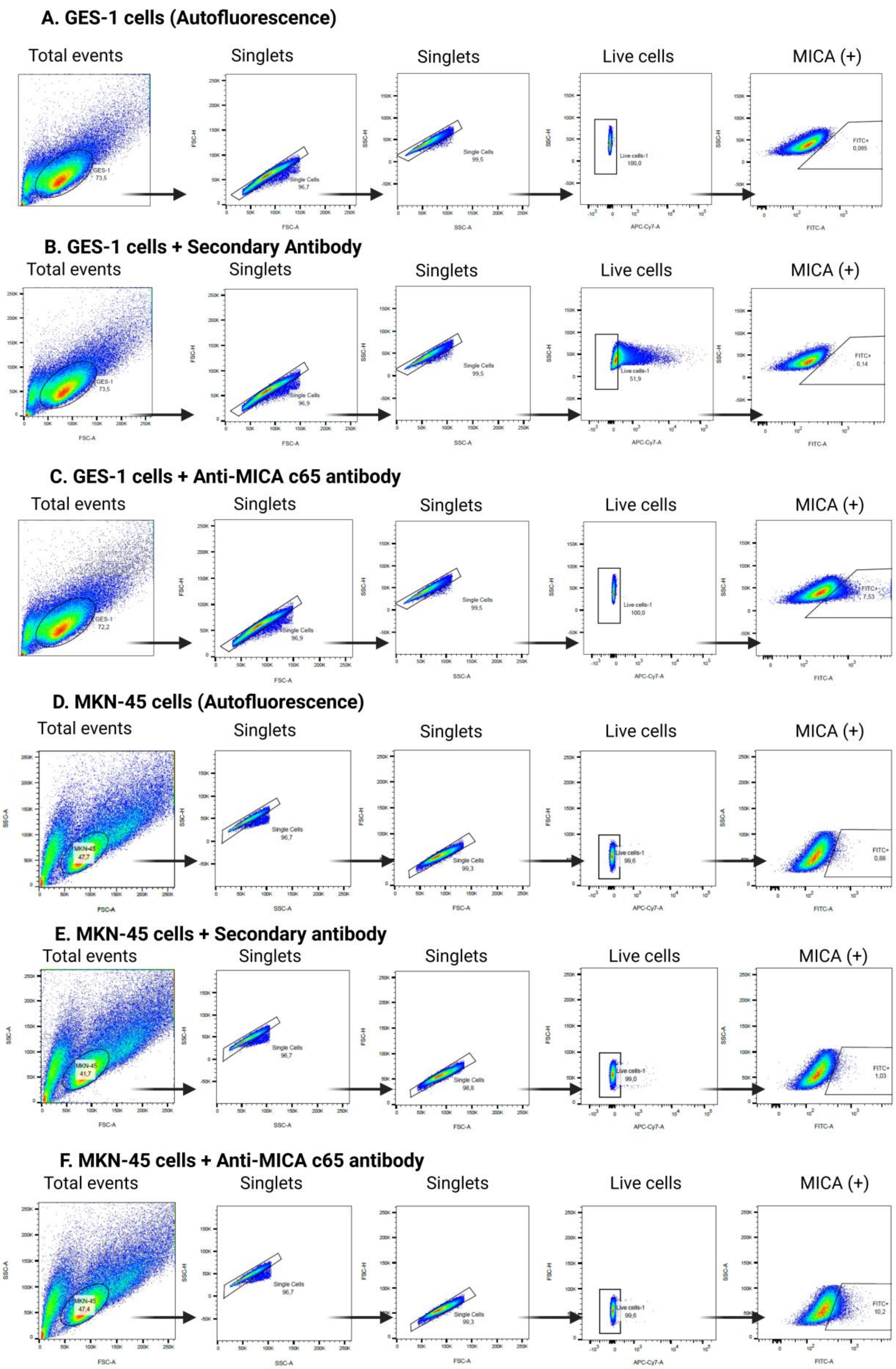
A. GES-1 cells (Autofluorescence)
Total events
Singlets
Singlets
Live cells
MICA (+)
B. GES-1 cells + Secondary Antibody
Total events
Singlets
Singlets
Live cells
MICA (+)
C. GES-1 cells + Anti-MICA c65 antibody
Total events
Singlets
Singlets
Live cells
MICA (+)
D. MKN-45 cells (Autofluorescence)
Total events
Singlets
Singlets
Live cells
MICA (+)
E. MKN-45 cells + Secondary antibody
Total events
Singlets
Singlets
Live cells
MICA (+)
F. MKN-45 cells + Anti-MICA c65 antibody
Total events
Singlets
Singlets
Live cells
MICA (+)

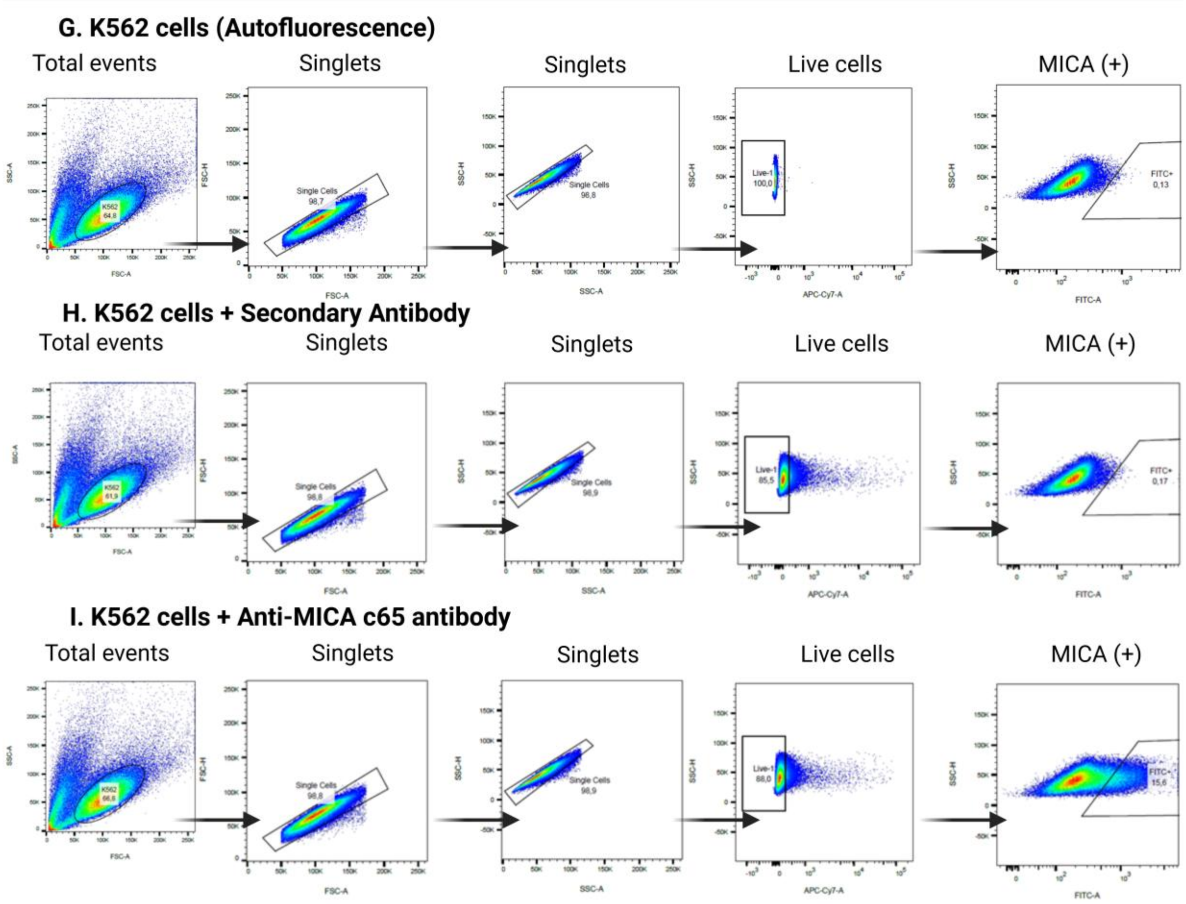

Supplementary Figure S4. Gating strategy for GES-1, MKN-45 and K562 cell lines and flow-cytometry binding of anti-MICA c65. (A, D, G) Unstained cells (autofluorescence). (B, E, H) Secondary antibody only (FITC-conjugated goat anti-human IgG [Fc-specific]), (C, F, I) Anti-MICA c65 antibody (1 µg). For all conditions, 0.5×10^6 cells were incubated at 4°C for 1 h as described in the Method section. Sequential gating was performed on total events (FSC-A vs SSC-A), followed by singlet discrimination (FSC-A vs FSC-H and SSC-A vs SSC-H), selection of live cells (Zombie NIR–negative), and final identification of the percentage of cells that are MICA-positive.

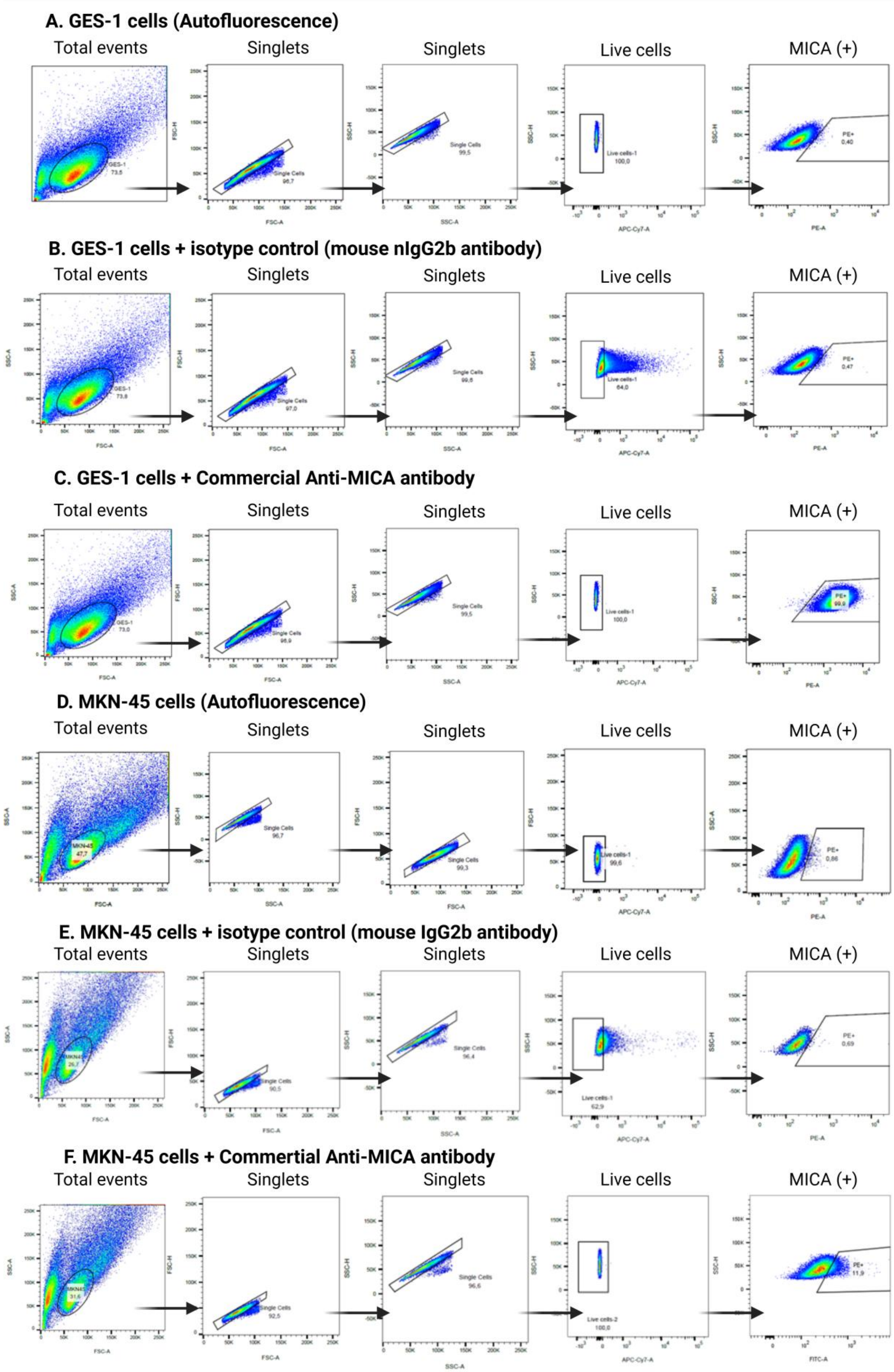
A. GES-1 cells (Autofluorescence)
B. GES-1 cells + isotype control (mouse nIgG2b antibody)
C. GES-1 cells + Commercial Anti-MICA antibody
D. MKN-45 cells (Autofluorescence)
E. MKN-45 cells + isotype control (mouse IgG2b antibody)
F. MKN-45 cells + Commertial Anti-MICA antibody
Total events
Singlets
Singlets
Live cells
MICA (+)

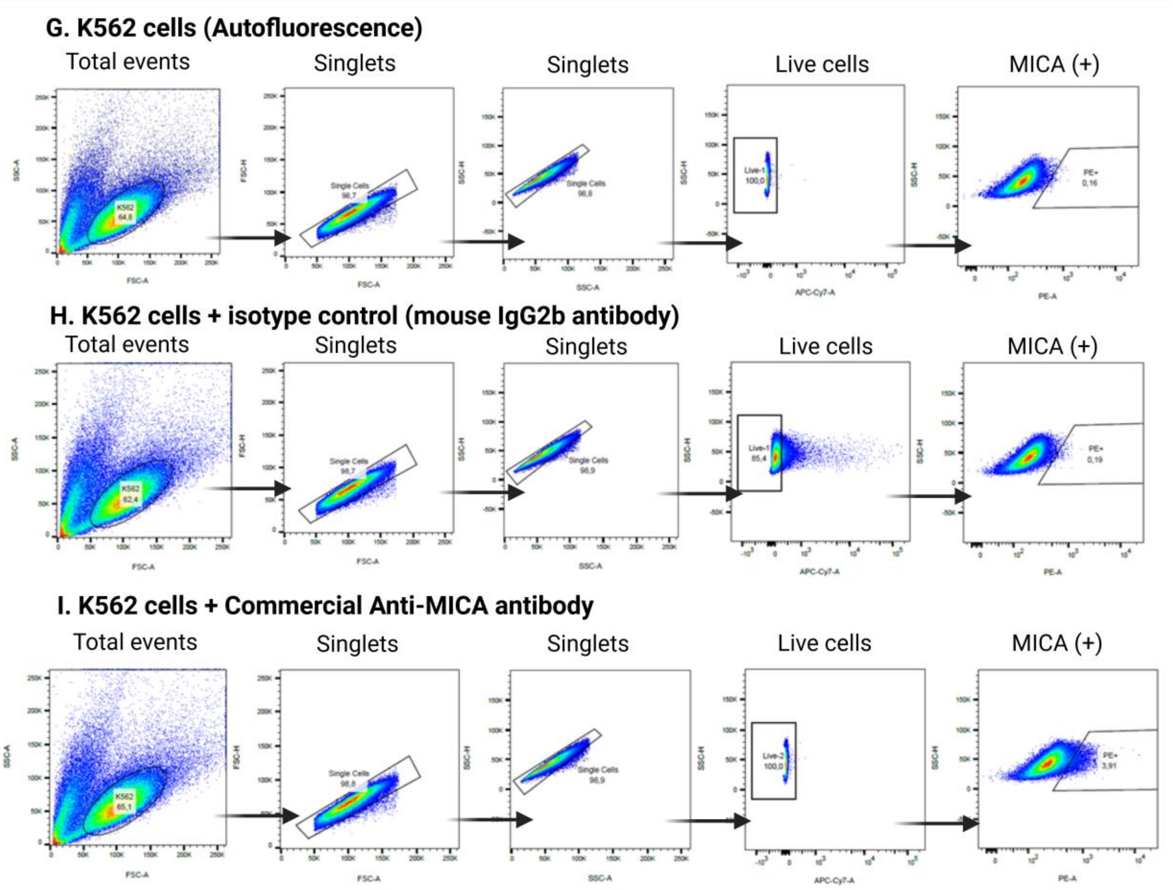

Supplementary Figure S5. Gating strategy for GES-1, MKN-45 and K562 cell lines and flow-cytometry binding of commercial anti-MICA antibody. (A, D, G) Unstained cells (autofluorescence). (B, E, H) Isotype control (mouse IgG2b) conjugated to phycoerythrin (PE). (C, F, I) Commercial mouse monoclonal anti-MICA antibody conjugated to PE. For all conditions, 0.5×10^6 cells were incubated at 4°C for 1 h as described in the Method section. Sequential gating was performed on total events (FSC-A vs SSC-A), followed by singlet discrimination (FSC-A vs FSC-H and SSC-A vs SSC-H), selection of live cells (Zombie NIR–negative), and final identification of the percentage of cells that are MICA-positive.

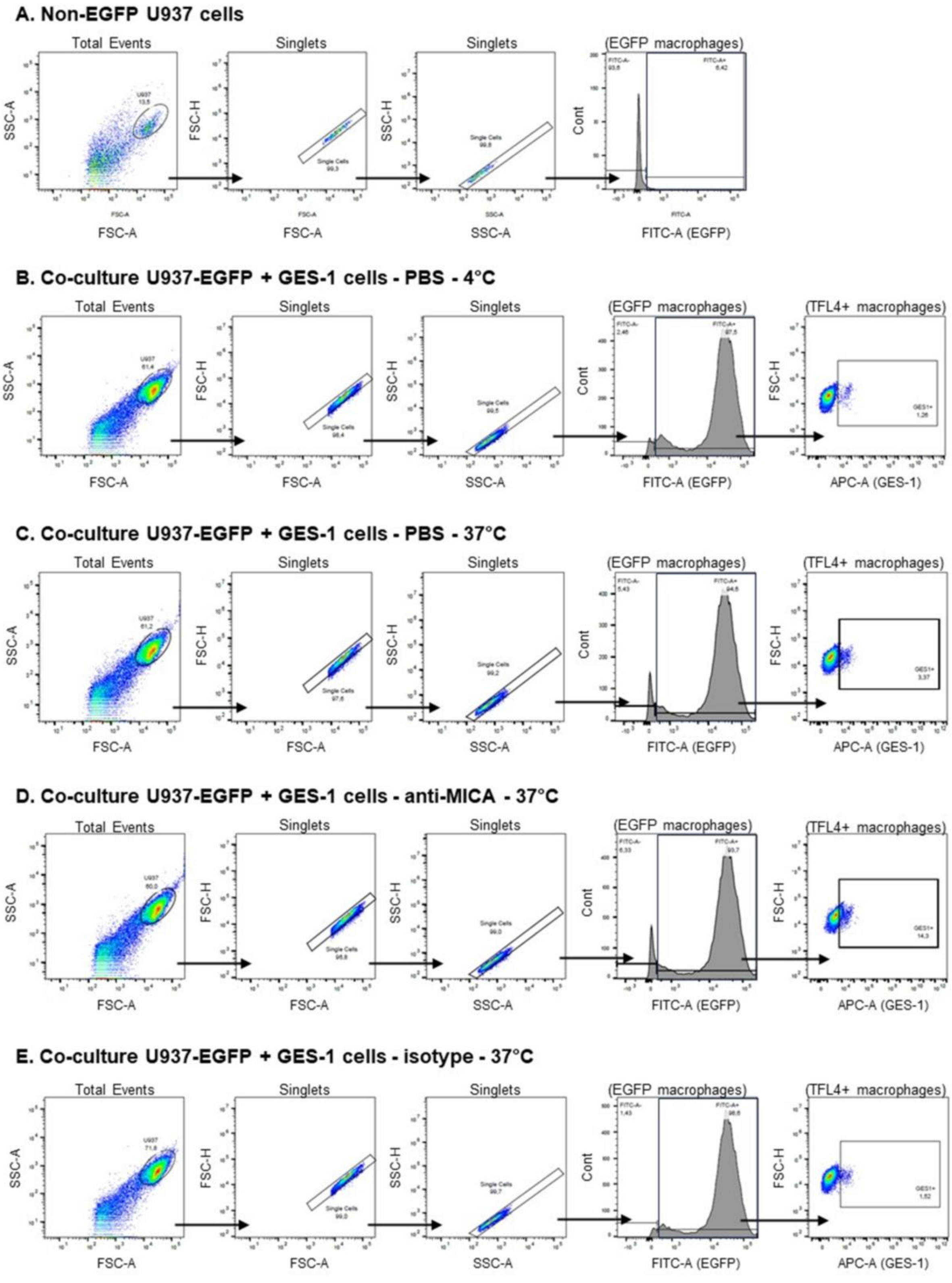
A. Non-EGFP U937 cells
Total Events
Singlets
Singlets
(EGFP macrophages)
SSC-A
FSC-H
SSC-H
Cont
FSC-A
FSC-A
SSC-A
FITC-A (EGFP)
B. Co-culture U937-EGFP + GES-1 cells - PBS - 4°C
Total Events
Singlets
Singlets
(EGFP macrophages)
(TFL4+ macrophages)
SSC-A
FSC-H
SSC-H
Cont
FSC-H
FSC-A
FSC-A
SSC-A
FITC-A (EGFP)
APC-A (GES-1)
C. Co-culture U937-EGFP + GES-1 cells - PBS - 37°C
Total Events
Singlets
Singlets
(EGFP macrophages)
(TFL4+ macrophages)
SSC-A
FSC-H
SSC-H
Cont
FSC-H
FSC-A
FSC-A
SSC-A
FITC-A (EGFP)
APC-A (GES-1)
D. Co-culture U937-EGFP + GES-1 cells - anti-MICA - 37°C
Total Events
Singlets
Singlets
(EGFP macrophages)
(TFL4+ macrophages)
SSC-A
FSC-H
SSC-H
Cont
FSC-H
FSC-A
FSC-A
SSC-A
FITC-A (EGFP)
APC-A (GES-1)
E. Co-culture U937-EGFP + GES-1 cells - isotype - 37°C
Total Events
Singlets
Singlets
(EGFP macrophages)
(TFL4+ macrophages)
SSC-A
FSC-H
SSC-H
Cont
FSC-H
FSC-A
FSC-A
SSC-A
FITC-A (EGFP)
APC-A (GES-1)

Supplementary Figure S6. Gating strategy for the quantification of antibody-dependent cell phagocytosis (ADCP). Representative flow cytometry plots illustrating the gating strategy used to identify phagocytic U937-derived macrophages in ADCP assays. (A) Non-EGFP U937 cells were used to define the $EGFP^+$ macrophage gate. (B–E) Co-cultures of U937-EGFP macrophages and TFL4-labeled GES-1 target cells under the indicated conditions: PBS at 4 °C (B), PBS at 37 °C (C), anti-MICA c65 antibody at 37 °C (D), and isotype control antibody at 37 °C (E). Sequential gating was performed on total events (FSC-A vs SSC-A), singlets (FSC-A vs FSC-H and SSC-A vs SSC-H), followed by identification of $EGFP^+$ macrophages. Phagocytic events were defined as $EGFP^+$ macrophages positive for APC (TFL4-labeled GES-1 cells).

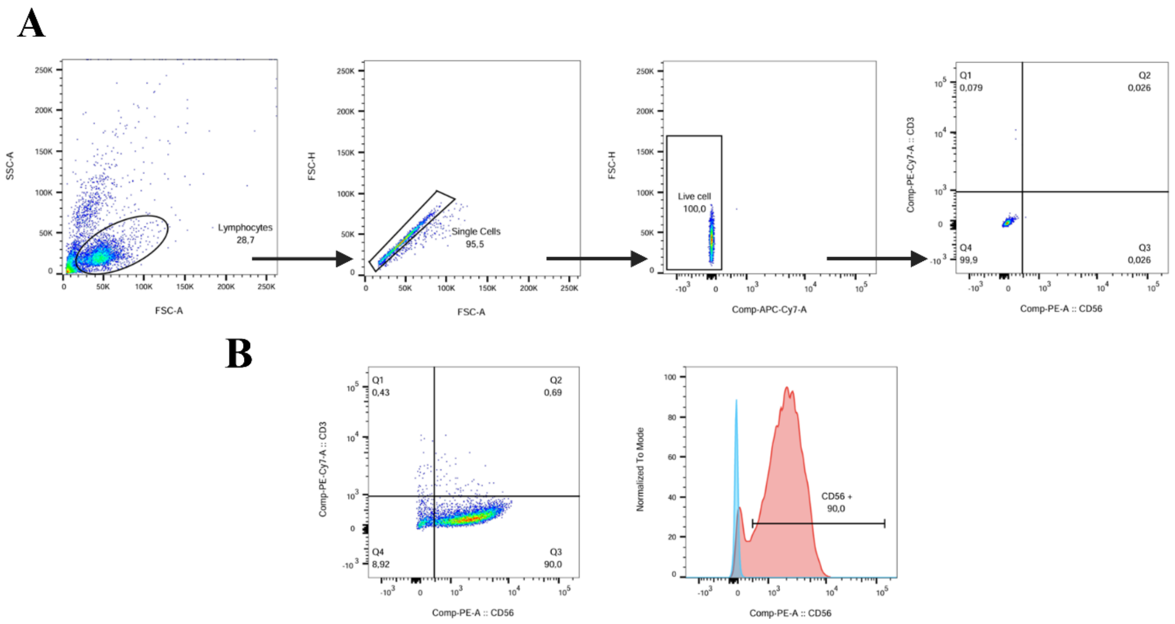

Supplementary Figure S7: Gating strategies used for primary human NK cells. (A) Gating sequence used to identify NK cells (CD56^{+}CD3^{-}). (B) Purified NK-cell fraction: CD3 vs CD56 plot and CD56 histogram showing the cell purity percentage.

A. CHO 008 cell autofluorescencs - untreated

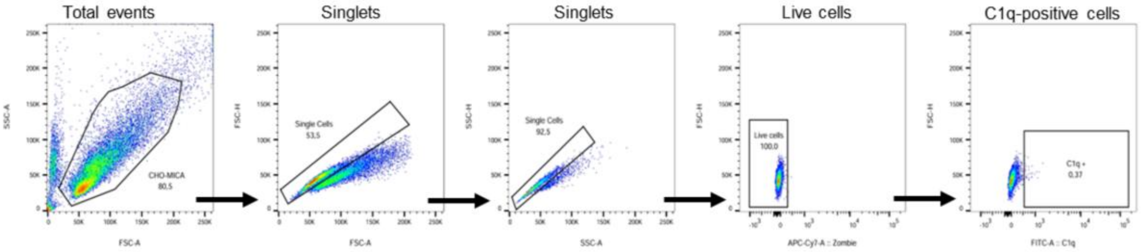

B. CHO 008 cell autofluorescencs - inactive serum

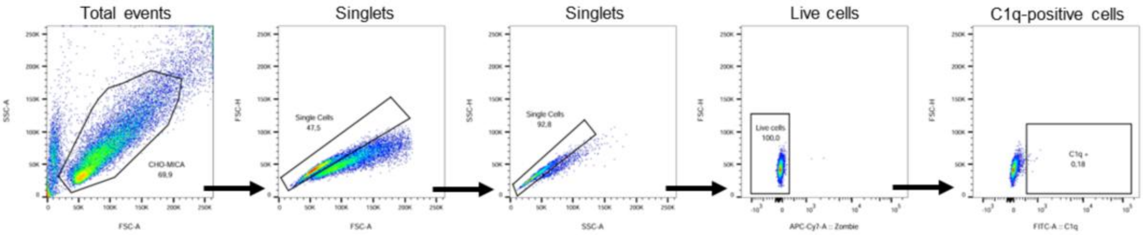

C. Inactivated serum + PBS treated group

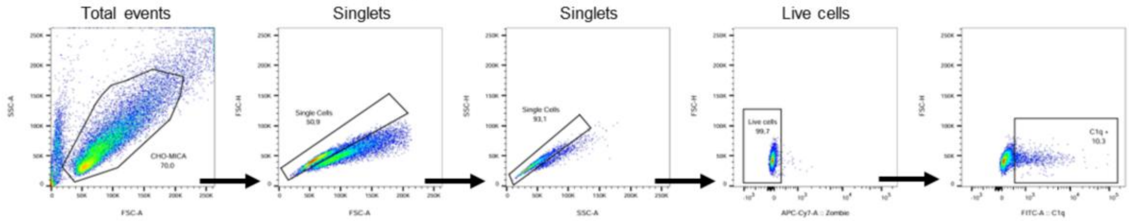

D. Inactivated serum + anti-MICA-c68 treated group

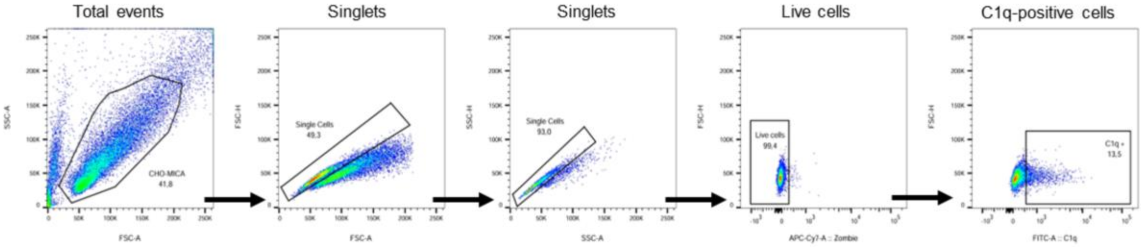

E. Inactivated serum + isotype treated group

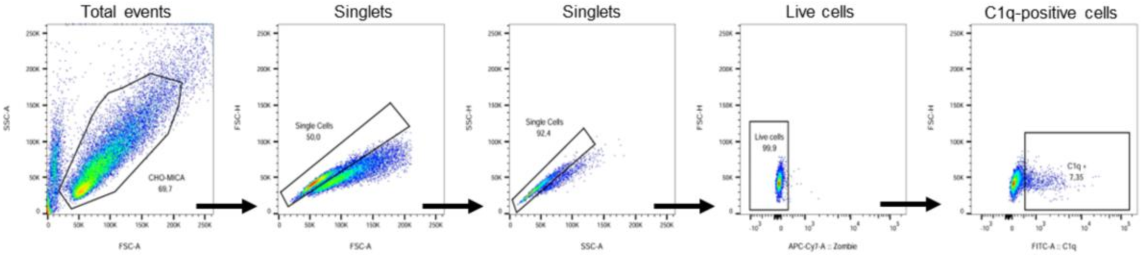

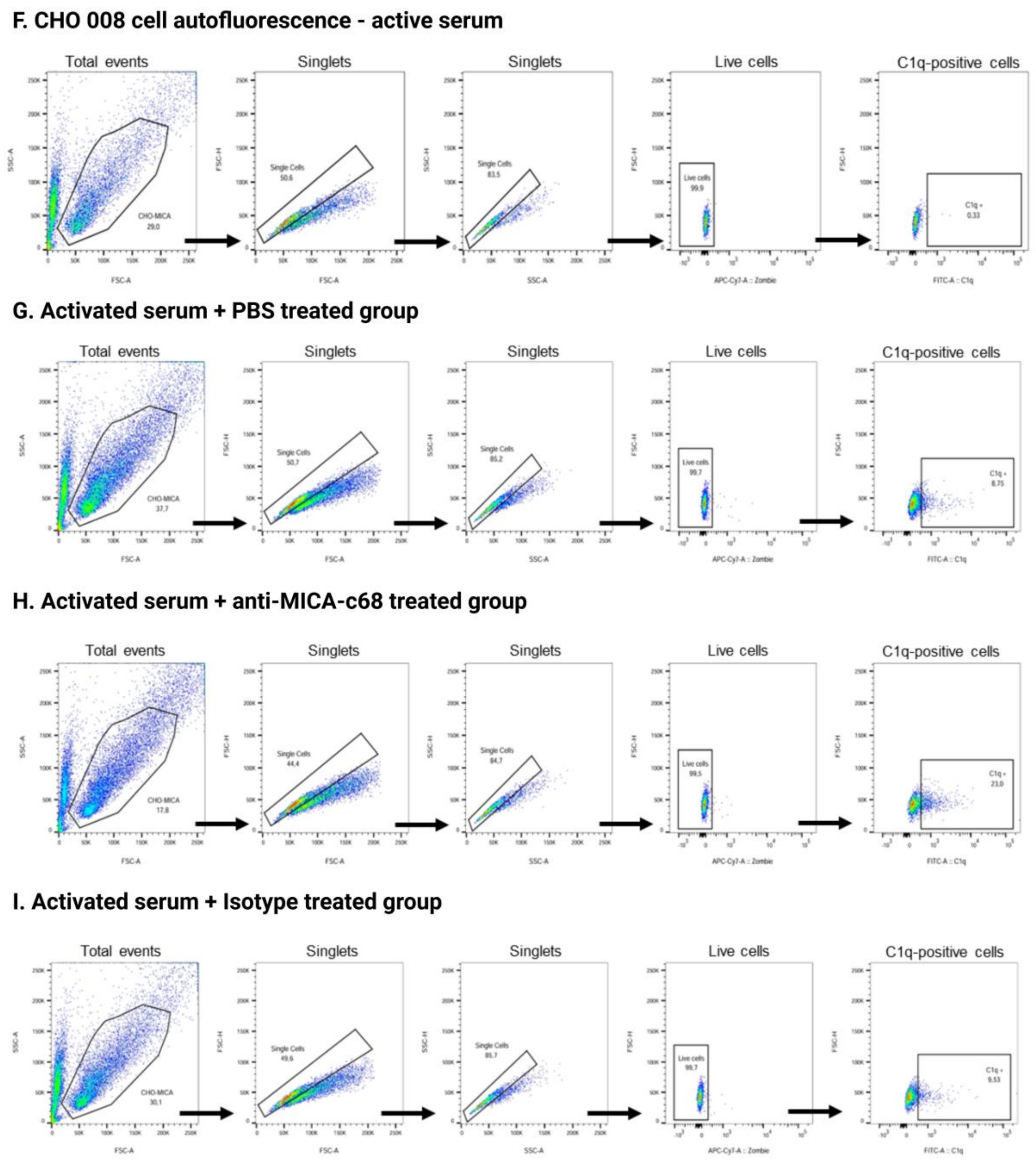

Supplementary Figure S8. Gating strategy for the analysis of C1q deposition on CHO 008 cells. Representative flow cytometry plots illustrating the gating strategy used to quantify C1q-positive events. (A) Untreated cells were used as autofluorescence control to define the basal gating strategy. (B) Cells treated with heat-inactivated human serum were used as autofluorescence control for heat-inactivated serum–treated conditions. (C–E) Cells incubated with heat-inactivated human serum under the indicated treatments: PBS (C), anti-MICA c65 antibody (D), and isotype control antibody (E). (F) Cells treated with active human serum were used as autofluorescence control for active serum conditions. (G–I) Cells incubated with active human serum under the indicated treatments: PBS (G), anti-MICA c65 antibody (H), and isotype control antibody (I). Sequential gating was performed on total events (FSC-A vs SSC-A), followed by singlet discrimination (FSC-A vs FSC-H and SSC-

A vs SSC-H), selection of live cells (Zombie NIR–negative), and final identification of C1q deposition, quantified as FITC–anti-human C1q–positive events.

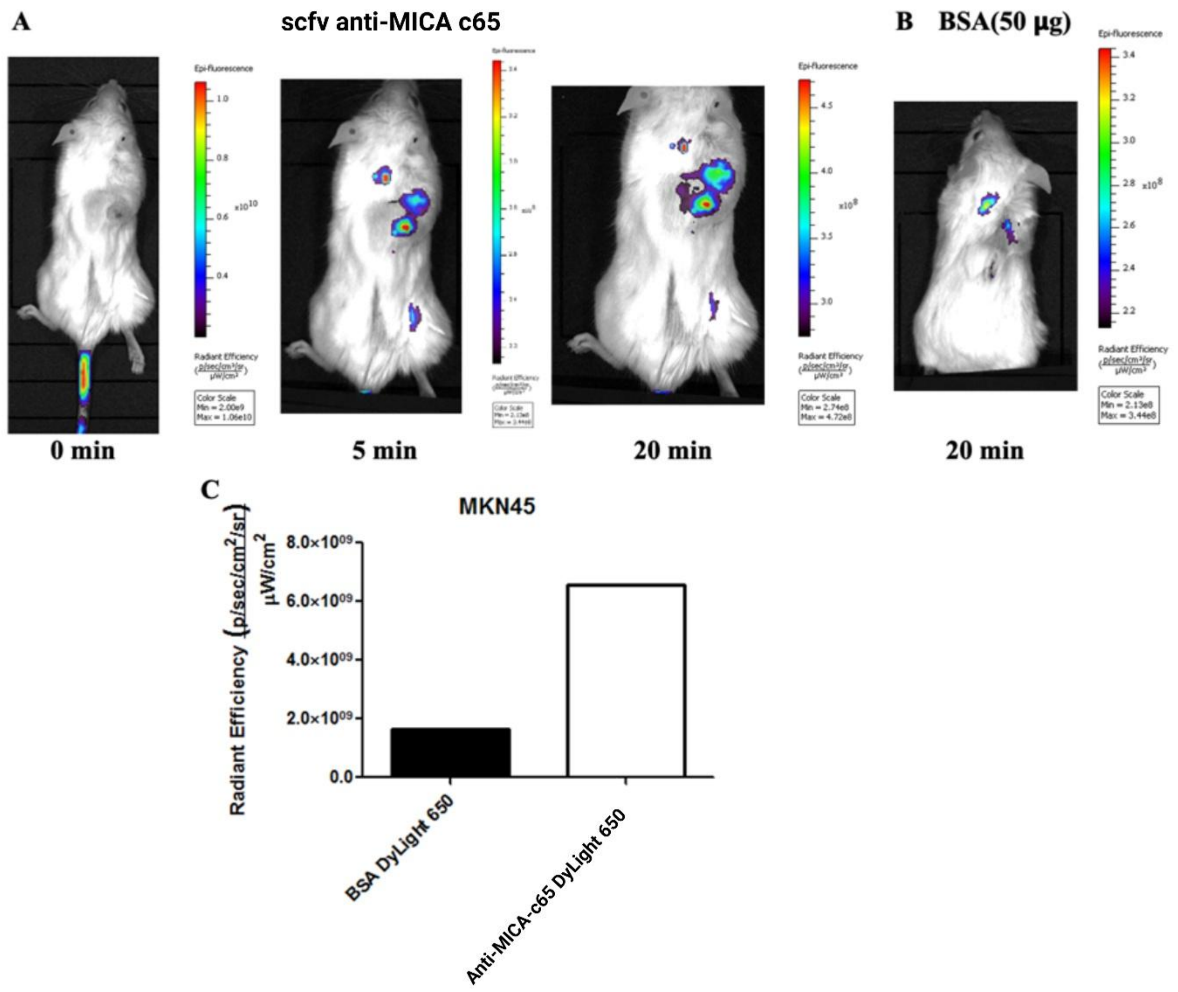

Supplementary Figure S9. In vivo tumor targeting of anti-MICA scFv-65 in a human MKN-45 xenograft model. Immunodeficient NOD/SCID mice bearing subcutaneous MKN-45 gastric tumors were injected intravenously with 50 µg of DyLight-650–conjugated anti-MICA scFv-c65. As a negative control, mice received 50 µg of DyLight-650–conjugated bovine serum albumin (BSA). Fluorescence emission was monitored using an IVIS Lumina II imaging system at 0, 5, and 20 min post-injection. (A) Representative images showing preferential accumulation of anti-MICA scFv-c65 at the tumor site over time. (B) Minimal tumor-associated signal in mice receiving BSA. (C) Quantification of radiant efficiency at 20 min post-injection demonstrating increased tumor-associated signal for anti-MICA scFv-c65 compared to BSA. Experiments were performed using the scFvc65 format to assess tumor targeting rather than therapeutic efficacy.
